# Sphingosine kinase 1/S1P receptor signaling axis is essential for cellular uptake of *Neisseria meningitidis* in brain endothelial cells

**DOI:** 10.1371/journal.ppat.1011842

**Published:** 2023-11-30

**Authors:** Ingo Fohmann, Alina Weinmann, Fabian Schumacher, Simon Peters, Agata Prell, Cynthia Weigel, Sarah Spiegel, Burkhard Kleuser, Alexandra Schubert-Unkmeir

**Affiliations:** 1 Institute for Hygiene and Microbiology, University of Würzburg, Würzburg, Germany; 2 Institute of Pharmacy, Pharmacology and Toxicology, Freie Universität Berlin, Berlin, Germany; 3 Department of Biochemistry and Molecular Biology and the Massey Cancer Center, Virginia Commonwealth University School of Medicine, Richmond, Virginia, United States of America; University of Virginia, UNITED STATES

## Abstract

Invasion of brain endothelial cells (BECs) is central to the pathogenicity of *Neisseria meningitidis* infection. Here, we established a key role for the bioactive sphingolipid sphingosine-1-phosphate (S1P) and S1P receptor (S1PR) 2 in the uptake process. Quantitative sphingolipidome analyses of BECs infected with *N*. *meningitidis* revealed elevated S1P levels, which could be attributed to enhanced expression of the enzyme sphingosine kinase 1 and its activity. Increased activity was dependent on the interaction of meningococcal type IV pilus with the endothelial receptor CD147. Concurrently, infection led to increased expression of the S1PR2. Blocking S1PR2 signaling impaired epidermal growth factor receptor (EGFR) phosphorylation, which has been shown to be involved in cytoskeletal remodeling and bacterial endocytosis. Strikingly, targeting S1PR1 or S1PR3 also interfered with bacterial uptake. Collectively, our data support a critical role of the SphK/S1P/S1PR axis in the invasion of *N*. *meningitidis* into BECs, defining a potential target for adjuvant therapy.

## Author summary

The gram-negative bacterium *Neisseria meningitidis* can cause live-threatening meningitis in humans after crossing the blood-brain barrier (BBB). During infection, *N*. *meningitidis* may invade brain endothelial cells as a first step to cross the BBB. The bioactive lipid Sphingosine 1-phosphate (S1P) is an important regulator of BBB integrity during health and disease. While interaction of S1P with S1P receptor (S1PR) 1 enhances barrier stability, activation of S1PR2 leads to barrier leakage. We found that *N*. *meningitidis* induces S1P release from brain endothelial cells in parallel with increased expression of S1PR2 and downstream activation of the epidermal growth factor receptor, indicating a shift in the S1PR signalling balance during infection. Moreover, we showed that inhibition of S1P production or S1PR2 signalling reduced bacterial uptake. Conversely, pharmacological activation of S1PR1 and 3 had an even higher protective effect against bacterial invasion, indicating that the S1P/S1PR axis is actively manipulated by *N*. *meningitidis* and thus highlighting potential drug targets to prevent meningococcal meningitis.

## Introduction

Sphingolipids are ubiquitous plasma membrane constituents, but can also act as bioactive molecules in eukaryotic cells [1]. Both functions are essential for cellular homeostasis and dysregulation can contribute to various conditions, such as Alzheimer’s disease, Parkinson’s disease, Multiple Sclerosis or infectious diseases [2]. Many pathogens, including bacteria, viruses and fungi, manipulate cellular membrane sphingolipids and metabolites to enhance their virulence [3, 4]. Some extracellular bacterial pathogens can use ceramide-enriched membrane platforms (CRPs) as an entry portal into various host cells [5–7]. Other microbes, such as obligate intracellular *Chlamydia trachomatis*, are able to capture host sphingolipids and incorporate them into their membranes [8]. The β-toxin-producing bacterium *Staphylococcus aureus* can actively target host sphingolipids by hydrolyzing sphingomyelin [9]. Furthermore, some bacteria e.g. members of the Bacteroidetes phylum are capable of synthesizing sphingolipid species in order to survive in hostile environments and improve replicative fitness [10].

Sphingosine-1 phosphate (S1P) is a sphingolipid metabolite that acts as a bioactive signaling molecule. S1P is formed by phosphorylation of sphingosine (Sph) by two isoforms of sphingosine kinases, SphK1 and SphK2 [11]. SphK1 activation can be regulated at the transcriptional, translational or post-translational level by phosphorylation and protein–protein interactions which can control both the activity and localization [12]. Phosphorylation of SphK1 at Ser-225 for example leads to the translocation of the kinase to caveolin-coated lipid rafts, where the enzyme encounters Sph and is thus stimulated to form S1P [13, 14]. While SphK1 is found in the cytoplasm [15], SphK2 is predominantly found in the nucleus, where S1P production can directly influence cellular transcription by manipulating telomerase reverse transcriptase or histone deacetylation [16, 17].

S1P is exported out of endothelial cells predominantly by the spinster homology protein 2 (Spns2) [18]. Extracellularly, S1P modulates a variety of cellular processes via binding to a family of five specific G protein-coupled receptors (S1PR1-5). Of these, S1PR1-3 are ubiquitously expressed, while S1PR4/5 expression is mostly restricted to hematopoietic cell types [19]. S1P is involved in regulating cardiovascular functions, neural development and immune cell transport [20]. S1P also modulates angiogenesis and stability of the vasculature [21]. This also applies for the brain microvasculature, where the balance between S1PR1/3- and S1PR2-signaling ensures the stability of the blood-brain-barrier (BBB) [22–25]. Recent findings attribute this effect not only to paracellular but also transcellular integrity [26].

*Neisseria meningitidis* (*N*. *meningitidis*) are commensal inhabitants of the human nasopharynx [27] and it is likely that most people are colonized at least once throughout their life time [28]. In rare cases *N*. *meningitidis* can cross the nasopharyngeal epithelial barrier to reach the bloodstream and cause severe septicemia and/or meningitis. The binding of bacteria to endothelial cells of the brain (BECs) and their invasion is a prerequisite for successful invasion of the cerebrospinal fluid, thus causing meningitis. *N*. *meningitidis* possesses a variety of surface virulence factors that contribute to BEC adhesion, including type IV pili (Tfp), the outer membrane protein OpcA [29], lipopolysaccharides and a number of so-called minor adhesion proteins [30]. Among them, Tfp play the most important role, enabling binding to endothelial receptor CD147/Basigin [31, 32] and β-adrenergic receptor [33] thereby mediating a signaling cascade that leads to the formation of membrane protrusions around the adherent bacteria and increases their ability to resist the mechanical forces generated by blood flow [34]. Tyrosine phosphorylation of host proteins occurs after *N*. *meningitidis* binding to BECs, leading to cytoskeletal remodeling with subsequent engulfment of the pathogen. Specifically, the two non-receptor tyrosine kinases, c-Src and focal adhesion kinase, as well as the epidermal growth factor receptor (EGFR) are activated to mediate bacterial uptake [35–37].

Of note, while *N*. *meningitidis* is not capable of synthesizing sphingolipids itself, it is able to modulate the sphingolipid content on BECs, depending on the expression of Tfp and OpcA [6, 7]. Mechanistically, *N*. *meningitidis* transiently activates acid sphingomyelinase (ASMase) and thereby induce increased ceramide (Cer) levels on the plasma membrane of BECs, leading to the formation of large CRPs that serve to reorganize and bundle receptor molecules and allow bacterial entry into the cells [6]. Cer levels increase only transiently under infection, consistent with further metabolization. However, nothing is yet known about the involvement of other sphingolipid metabolic enzymes in *N*. *meningitidis* infection.

Here, we profiled the sphingolipid metabolome using LC-MS/MS measurements to provide a time-resolved image of sphingolipid metabolites in BECs in response to infection with *N*. *meningitidis*. We observed a significant increase of S1P release that was due to enhanced abundance of SphK1 transcript as well as enzyme activity in response to *N*. *meningitidis* infection. Increased activity was found to be dependent on Tfp interaction with its cognate receptor CD147. We showed that *N*. *meningitidis* infection increased *S1PR2* expression, leading to the activation of the S1P-S1PR2-EGFR axis and ultimately to bacterial uptake. Activation of S1PR1+3 however reduced bacterial uptake. This study demonstrates tight regulation of the S1PR2 and S1PR1+3 signaling on BECs disrupted during *N*. *meningitidis* infection by a novel criss-cross transactivation mechanism involving the three individual receptor systems CD147, S1PR2 and EGFR and illustrates the possible therapeutic potential of targeting this signaling cascade in meningococcal meningitis.

## Results

### *N*. *meningitidis* modulates the sphingolipid metabolome in BECs

**Fig 1:**
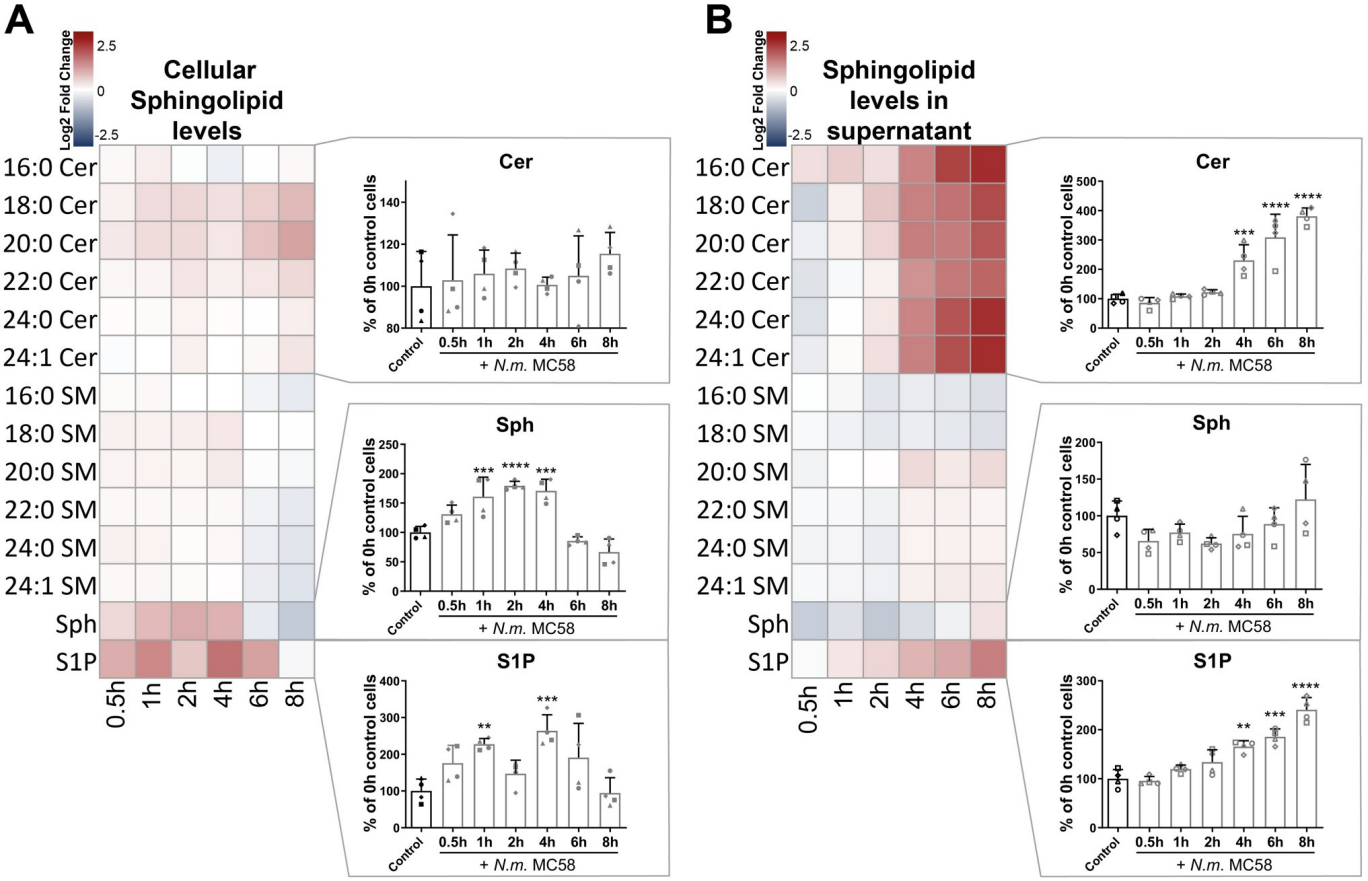
*N*. *meningitidis* induces elevation of sphingosine 1-phosphate levels in hCMEC/D3s. (A+B) hCMEC/D3 were infected with *N*. *meningitidis* strain MC58 at a MOI of 100, and sphingolipid levels were quantified at indicated time points by LC-MS/MS in cell lysates (cellular, A) or in supernatants (extracellular, B). Ceramide (Cer), Sphingosine (Sph), Sphingomyelin (SM) and Sphingosine 1-phosphate (S1P). Heatmap representing the log2 fold-change values of measured sphingolipid species between infected and uninfected control cells (control). Graphs/Diagrams show percentage changes of Cer (upper panel), Sph (middle panel) and S1P (lower panel) relative to uninfected control cells. The values of fold changes are indicated by the coloured scale, with deep red indicating a value>1 and blue indicating a value <1. Data are the means of four biological replicates ± SD. ** p < 0.01, *** p < 0.001, **** p < 0.0001 as calculated with one-way ANOVA followed by Dunnett‘s post-hoc test compared to uninfected control cells.

To examine whether in addition to ASMase, infection of BECs with *N*. *meningitidis* also regulated the activity of other sphingolipid metabolic enzymes, we first conducted a sphingolipid metabolome time-resolved survey using LC-ESI-MS/MS measurements. Targeted quantitative measurement of sphingolipids was performed at the indicated time points in brain microvascular endothelial hCMEC/D3 cells either left uninfected (control) or infected with *N*. *meningitidis* strain MC58 (Fig 1A) or in their supernatants (Fig 1B).

Data analysis showed a moderate increase in cellular Cer in hCMEC/D3 and the differences did not reach statistical significance (Fig 1A). When comparing the extracellular levels of Cer, enhanced levels in Cer were observed in the supernatants of infected hCMEC/D3 (Fig 1B), suggesting that excessively produced Cer was not fully retained in the plasma membrane. Interestingly, increase of major Cer species 16: 0, 24:0 and 24:1 was mostly observed extracellularly, whereas the low abundant species Cer species 18:0 and 20:0 Cer were prominently increased in both compartments (Fig 1, Heatmaps). Cellular Sphingomyelin (SM) levels were non-significantly reduced over 8h infection time course (Fig 1A, Heatmap), most likely due to the turnover to Cer, whereas the extracellular SM levels were only marginally increased (Fig 1B, Heatmap).

Cellular Sphingosine (Sph) levels increased in response to infection with *N*. *meningitidis* MC58, peaking 2h post-infection (p.i.) (Fig 1A), whereas the Sph levels in the supernatant did not change (Fig 1B). Importantly, cellular S1P significantly increased reaching a peak 4h p.i. and returned to basal levels by 8h (Fig 1A). Moreover, S1P levels increased continuously in the supernatant, reaching 240% of basal S1P levels over the 8h infection time course (Fig 1B).

### *N*. *meningitidis* transiently activates SphK1 expression and activity

**Fig 2:**
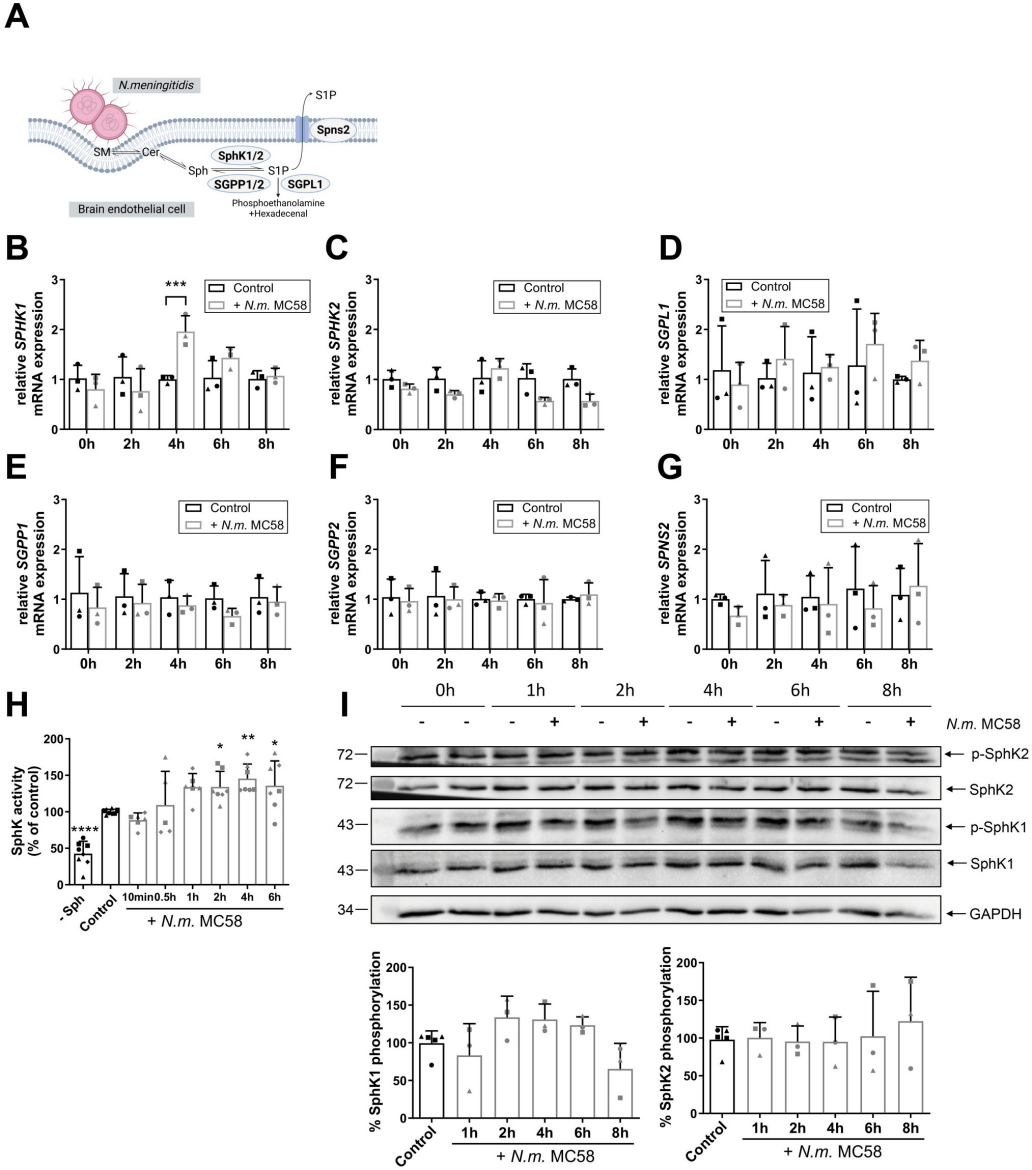
*N*. *meningitidis* transiently activates SphKs. (A) Schematic representation of the regulation of S1P homeostasis by sphingosine kinase 1/2 (SphK1/2), S1P phosphatase 1/2 (SGPP1/2), S1P lyase (SGPL1) and S1P transporter Spinster 2 (Spns2). (B-G) Fold change in expression of *SPHK1* (B), *SPHK2* (C), *SGPL1* (D), *SGPP1* (E), *SGPP2* (F) and *SPNS2* (G) in hCMEC/D3s infected with *N*. *meningitidis* MC58 for an 8h infection time course normalized to 18S rRNA measured by qPCR. Data represent mean ± SD of 3 independent experiments measured in duplicates. Comparison to control for each time point using multiple t-test and p-value adjustment with Holm-Sidak correction. ***p<0.001 vs. respective control. (H) SphK enzymatic activity measured in hCMEC/D3s infected with *N*. *meningitidis* MC58 for a 6 h infection time in comparison to uninfected control cells as determined by ATP depletion assay. Uninfected control cells without addition of SphK substrate Sph (‘-Sph’) were used to detect non-specific ATP consumption. Graph represents the mean ± SD of at least three independent experiments with biological duplicates and is reported as activity normalized to control cells. One-Way ANOVA followed by by Dunett’s post-hoc test. *p<0.05, **p<0.01, ****p<0.0001 vs. control. (I) hCMEC/D3s were infected with *N*. *meningitidis* MC58 for 8h infection time course and both total protein and/or phosphorylated form of SphK1 (pSer-225) and SphK2 (pThr-578) were assessed by Western blot. Representative immunoblot is shown. Graphs represent the mean ± SD of three independent experiments and reported as phosphorylated form relative to total target protein normalized to control cells. One-Way ANOVA showed no significance. Related to S1 Fig.

To determine whether S1P metabolic enzymes (Fig 2A) contribute to the increase of S1P levels we measured the levels of their mRNA transcripts in hCMEC/D3s infected with *N*. *meningitidis* MC58 over 8h infection time course using quantitative real time PCR (qRT-PCR). SphK1 mRNA levels were significantly upregulated in infected hCMEC/D3 at 4h p.i. compared to uninfected control cells (Fig 2B), while SphK2 transcript levels were not changed and even decreased at 6h and 8h p.i. (Fig 2C). No significant changes in mRNA levels of degradative enzymes S1P phosphatase 1 and 2 (SGPP1, SGPP2) and S1P lyase (SGPL1) were detected (Fig 2D–2F). Likewise, *N*. *meningitidis* infection did not affect the expression of Spns2, the transporter mainly responsible for the secretion of S1P from endothelial cells (Fig 2G) [18].

We next measured SphK activity in BECs. Infection with *N*. *meningitidis* significantly increased SphK activity by 2h and remained elevated thereafter (Fig 2H), thus demonstrating that *N*. *meningitidis* infection alters abundance of SphK1 transcript as well as enzymatic activity.

Phosphorylation of SphK1 at Ser-225 induces SphK1 activation through specific interaction with phosphatidylserine resulting in recruitment to the plasma membrane [14]. Infection of hCMEC/D3 with *N*. *meningitidis* MC58 slightly increased phosphorylation levels of SphK1 at Ser-225 in whole cell lysates, albeit not significantly (Fig 2I). We therefore aimed to detect p(Ser-225)-SphK1 translocation by purifying plasma membranes from hCMEC/D3s, which were infected with *N*. *meningitidis* MC58 for 4h or left uninfected. Western Blot analysis showed, that SphK1 protein levels were increased and p(Ser-225)-SphK1 was only detectable in the plasma membrane fraction after infection, indicating increased phospho-activation and translocation of SphK1 upon meningococcal infection (S1B Fig). We also examined a possible activation of SphK2 through the ERK1 phosphorylation site Thr-578 [38]. However, p(Thr-578)-SphK2 levels were not changed in *N*. *meningitidis*-infected hCMEC/D3s (Fig 2I).

### *N*. *meningitidis* Tfp binding to CD147 contributes to SphK activity

**Fig 3:**
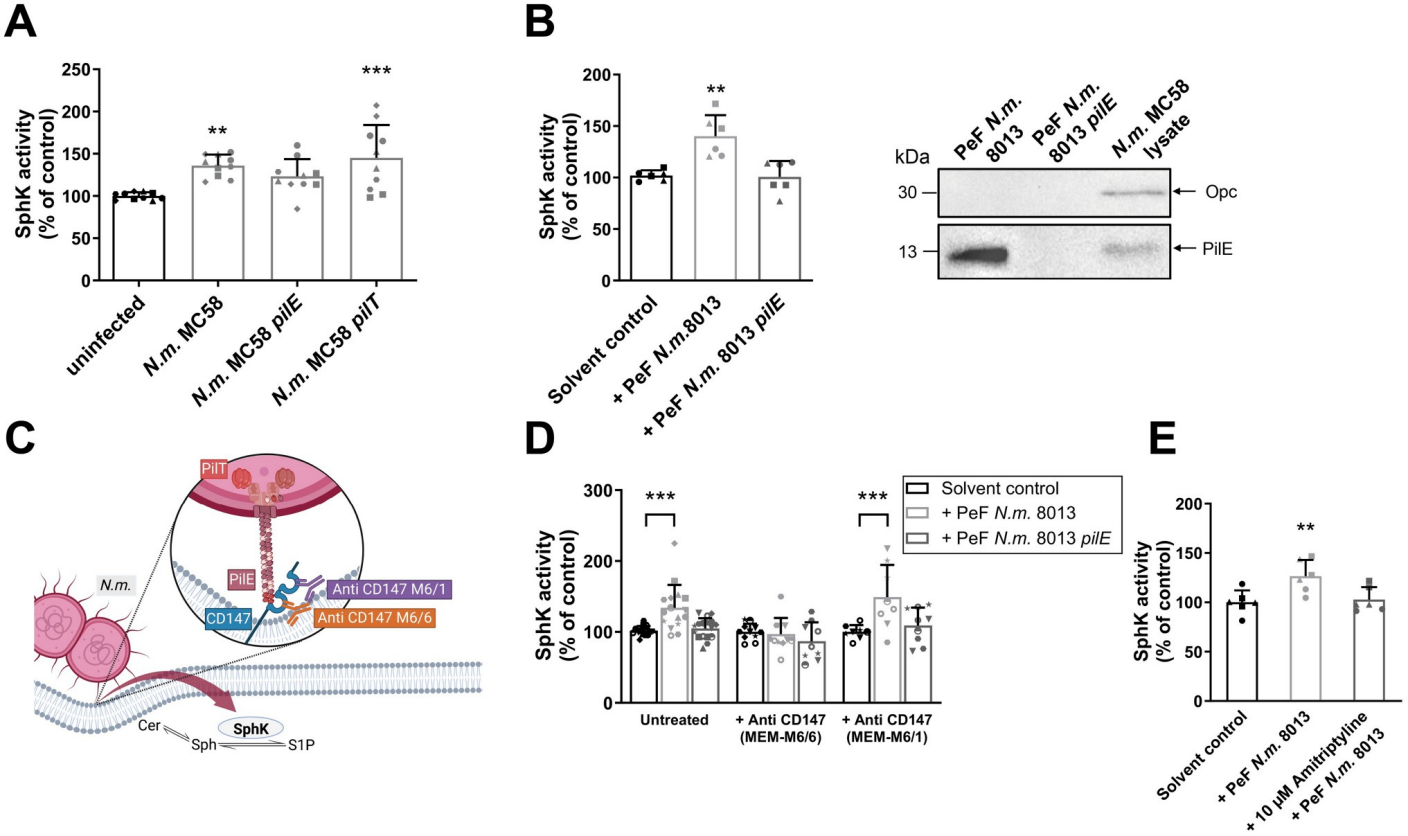
Tfp binding to CD147 contributes to SphK activity. (A) SphK enzymatic activity measured after 4h infection of hCMEC/D3s with wildtype strain *N*. *meningitidis* MC58, isogenic non-piliated *pilE* mutant or isogenic *pilT* mutant, lacking the Tfp retraction machinery. Data are presented as means ± SD, n = 5, N = 2. One-way ANOVA followed by Dunnett‘s post-hoc test.** p < 0.01, *** p < 0.001 compared with uninfected control cells. (B) SphK enzymatic activity in hCMEC/D3s stimulated with pilus–enriched fractions (PeF) of highly-piliated *N*. *meningitidis* serogroup C strain 8013/clone12 or isogenic *pilE* mutant or TBS as solvent control for 10 min. Data are means ± SD, n = 3, N = 2 experiments. ** p < 0.01, ANOVA followed by Dunnett‘s post-hoc test. (B, right) Assessment of pilus enrichment in PeF of strain 8013/clone12 compared to mutant strain 8013/clone12 *pilE* and bacterial lysate of *N*. *meningitidis* MC58. Additionally, lack of OpcA expression was controlled. (C) Schematic outline of Tfp interaction with CD147. CD147 is a single pass membrane protein with two extracellular Ig-like domains. The N-terminal IgC2 domain is recognized by the MEM-M6/1 antibody, the membrane proximal IgI domain is the competitive binding site for MEM-M6/6 and Tfp of *N*. *meningitidis*. (D+E) SphK activity in hCMEC/D3s preincubated for 30 min with 10 μg mL^**−1**^ of antibodies targeting the N-terminal Ig domain of CD147 (MEM-M6/1) or the C-terminal Ig domain of CD147 (MEM-M6/6) (D) or 10 μM Amitriptylin (E), quantified following a 10 min PeF treatment. Mean ± SD, *n* ≥ 3, N = 2. One-way ANOVA followed by Dunnett‘s post-hoc test. ** p < 0.01, *** p < 0.001.

Type IV pili (Tfp) of *N*. *meningitidis* trigger translocation and activation of ASMase in BECs [7]. To examine whether Tfp also contribute to SphK activation, hCMEC/D3s were infected with wildtype strain *N*. *meningitidis* MC58 (Fig 3A), mutant strain MC58 *pilE*, lacking the major constituent of Tfp PilE, or mutant strain MC58 *pilT*, lacking the pilus retraction machinery [39], leading to a hyper-piliated state of the Δ*pilT* mutant [40]. While MC58 *pilE* mutant had no effect, the hyper-piliated MC58 *pilT* mutant elicited a significant increase of SphK activity (Fig 3A). To further corroborate the role of Tfp, we next purified pili from *N*. *meningitidis strain* 8013/clone12 [41], a meningococcal isolate that is highly piliated but lacks Opa and OpcA expression (Fig 3B). hCMEC/D3s were treated with pilus-enriched fractions (PeF) and PeF from an isogenic pilus-deficient mutant served as control. PeF from wildtype strain 8013/12 induced a significant increase of SphK activity 10 min after treatment, whereas PeF from the pilus-deficient mutant 8013/12 *pilE* did not (Fig 3B).

Attachment of Tfp to the endothelium is mediated by binding to the proximal extracellular immunoglobulin (IgG) domain of cellular surface receptor CD147 [32]. To investigate, whether binding to CD147 leads to SphK activation, we tested two antibodies specific for CD147, MEM-M6/1 and MEM-M6/6, which bind to the extracellular N-terminal and the C-terminal Ig domains of CD147, respectively (Fig 3C) [31]. Blockade of the membrane-distant domain with anti-CD147 Ab (MEM-M6/1) did not interfere with SphK activation, whereas competitive blockage of the binding domain with anti-CD147 Ab (MEM-M6/6) prevented PeF-induced SphK activation (Fig 3D). In addition, pre-treatment with ASMase inhibitor Amitriptyline [6] similarly prevented PeF-induced SphK activation (Fig 3E). Taken together, these results indicate, that *N*. *meningitidis* activates SphK in hCMEC/D3s potentially downstream of ASMase activation and dependent on the important virulence factor Tfp, and activation implicates CD147 as an important receptor.

### *N*. *meningitidis* induces S1PR2-mediated EGFR activation to enable its uptake by hCMEC/D3s

**Fig 4:**
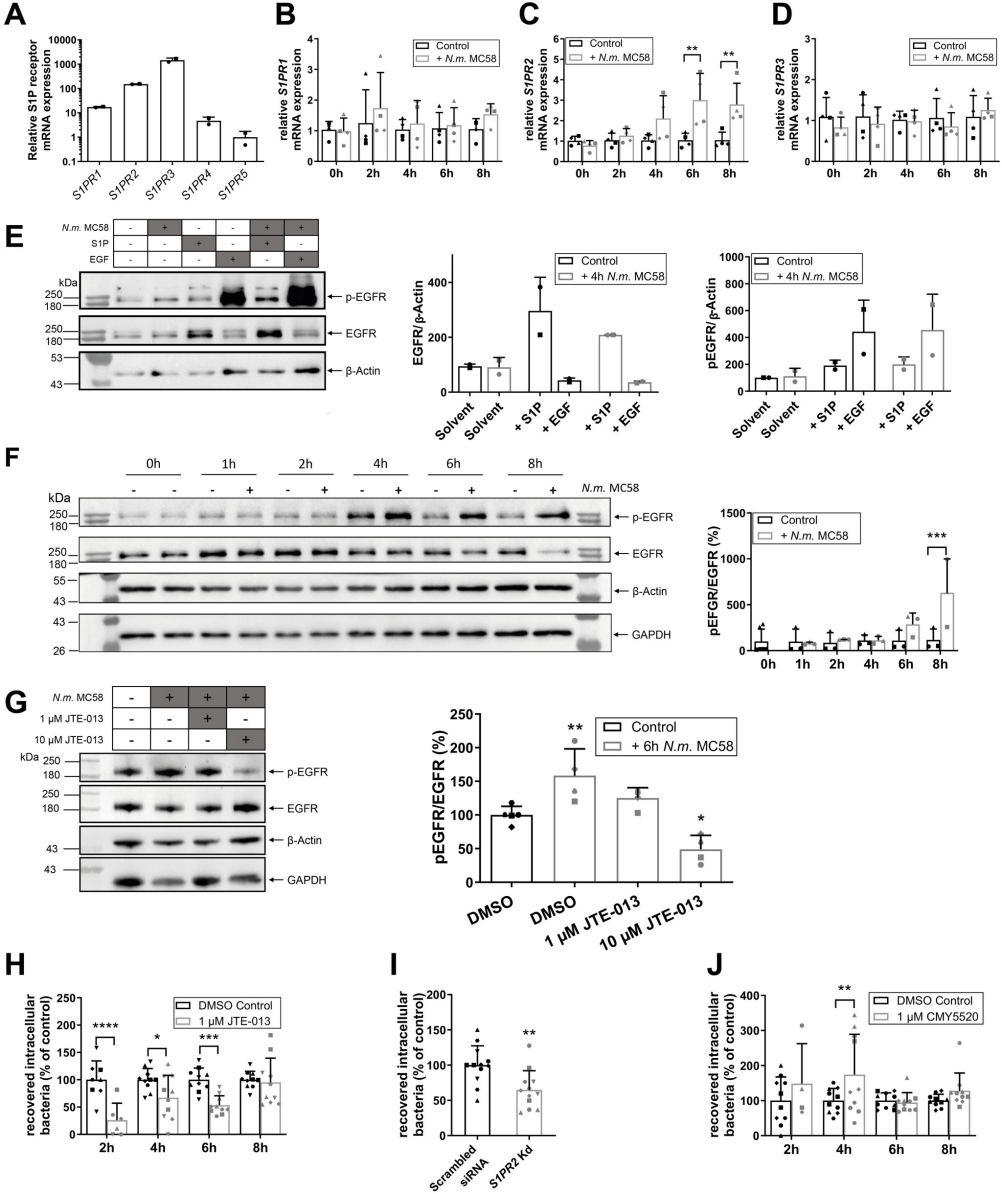
*N*. *meningitidis* induces S1PR2-mediated EGFR activation to enable invasion into hCMEC/D3s. (A) Relative mRNA expression of S1P receptors *S1PR1*, *S1PR2*, *S1PR3*, *S1PR4* and *S1PR5* in hCMEC/D3s determined by quantitative RT-PCR. Data are normalized to 18S rRNA relative to *S1PR5* mRNA expression. (B) *S1PR1* expression, (C) *S1PR2* expression, (D) *S1PR3* expression in hCMEC/D3s infected with *N*. *meningitidis* MC58 for 8h. mRNA expression levels were analyzed by qRT-PCR with normalization to 18S rRNA relative to uninfected control cells. Data are shown as mean ± SD, *n* = 4 experiments measured in duplicates. Comparison to control for each time point using multiple t-test and p-value adjustment with Holm-Sidak correction. ** p < 0.01 vs. respective control. (E) Determination of EGFR protein levels and phosphorylation at Tyr-845 (p-EGFR) in hCMEC/D3 cell lysates by Western blot analyses. hCMEC/D3s were stimulated with 1 μM S1P or 25 ng/mL EGF for 15 min, or additionally infected with *N*. *meningitidis* MC58. Cells were lysed with RIPA buffer, and cell lysate proteins were resolved with SDS-PAGE. Tyrosine phosphorylation of EGFR was analyzed using phospho-specific EGFR antibody (Tyr-845). Stripped membranes were blotted with EGFR antibody. A representative Western blot is shown. Densitometric analyses show data of two independent experiments. (F) Determination of EGFR expression and phosphorylation at Tyr-845 (p-EGFR) in hCMEC/D3s infected for 8h by Western blot and assessment by densiometric analyses. Data represent means ± SD, n = 3. One-way ANOVA followed by Dunnett’s post-hoc test, ***p<0.001. (G) Assessment of pharmacological inhibition of S1PR2 on EGFR protein levels and phosphorylation at Tyr-845. hCMEC/D3s were either pretreated with S1PR2/4 antagonist JTE-013 or solvent control (DMSO) and infected with *N*. *meningitidis* MC58 or left uninfected. Cell lysates were analyzed by Western blot and subsequent densiometric analysis. Data represent means ± SD, n = 4. One-way ANOVA followed by Dunnett’s post-hoc test, *p<0.05, **p<0.01. (H, I, J) Assessment of siRNA targeted knockdown of S1PR2 or pharmacological modulation of S1PR2 on bacterial invasion into BECs. hCMEC/D3 were pre-treated with (H) JTE-013 or (J) CYM5520 for 30 min or (I) with siRNA specific for S1PR2 for 72h. Cells were subsequently infected with *N*. *meningitidis* MC58 for 8h. Numbers of viable intracellular bacteria were determined by gentamicin protection assay and normalized to untreated control cells (DMSO control or scrambled siRNA for each time point). Data are shown as mean ± SD; n ≥ 4. Unpaired students t-test (I) or multiple t-test and p-value adjustment with Holm-Sidak correction. * p < 0.05, ** p < 0.01, *** p < 0.001, **** p < 0.0001. Absence of cytotoxicity, knockdown efficiency and bacterial growth were verified as shown in S2 and S4 Figs.

The sphingolipid metabolome survey revealed a continuous increase of extracellular S1P released from hCMEC/D3s upon infection with *N*. *meningitidis* (Fig 1A and 1B). To establish the functional role of increased extracellular S1P and its receptors, we first determined expression of *S1PR1-5* in hCMEC/D3. *S1PR1-3* were highly expressed in uninfected hCMEC/D3 cells, with negligible expression levels of *S1PR4* and *S1PR5* (Fig 4A). To evaluate the effects of *N*. *meningitidis* infection on the expression of S1PRs, hCMEC/D3s were infected for a period of 8h. Neither the mRNA levels of *S1PR1* nor *S1PR3* were affected by infection with *N*. *meningitidis* MC58 (Fig 4B and 4D). By contrast, mRNA levels of *S1PR2* in hCMEC/D3 increased significantly (Fig 4C).

Since previous studies have shown that uptake of *N*. *meningitidis* by BECs is mediated by activation of EGFR through phosphorylation at Tyr-845 [35] and a cross-talk between EGFR and S1P signaling has been reported [42], we sought to investigate a possible link between S1P, S1PR2 and the activation of EGFR in BECs in response to *N*. *meningitidis* infection. S1P treatment increased protein levels and Tyr-845 phosphorylation of EGFR (Fig 4E). Infection of hCMEC/D3 with *N*. *meningitidis* for 4h did not further increase S1P or EGF mediated phosphorylation of EGFR (Fig 4E). Only longer infection induced EGFR phosphorylation, which increased until 8h p.i. (Fig 4F). EGFR protein levels however did not change in response to infection and even declined at 8h p.i., suggesting a partial turnover of EGFR at later time points of infection following EGF-mediated EGFR activation [43].

To further test, whether S1PR2 is involved in EGFR phosphorylation, hCMEC/D3 were treated with JTE-013, a S1P receptor antagonist, highly selective for S1PR2 [44]. JTE-013 exhibited a dose-dependent inhibition of phosphorylation of EGFR measured in hCMEC/D3 cell lysates infected with *N*. *meningitidis* for 6h in a dose dependent manner. While treatment with 1 μM JTE-013 greatly prevented infection-induced EGFR phosphorylation, 10 μM JTE-013 reduced EGFR phosphorylation even below activation levels of uninfected control cells (Fig 4G).

EGFR activation has been linked to c-Src activation [35] which regulates cytoskeletal rearrangement known to be essential for *N*. *meningitidis* uptake in BECs [37]. We therefore determined the role of S1PR2 in *N*. *meningitidis* invasion of hCEMC/D3 using JTE-013 at a concentration of 1 μM (S4 Fig). Only 1 μM was tested, as it was recently reported that treatment with 10 μM JTE-013 has significant off-target effects on SphKs [45] and 1 μM prevented significant EGFR phosphorylation 6h p.i., without affecting *N*. *meningitidis* adhesion (S4C Fig). JTE-013 treatment was sufficient to inhibit *N*. *meningitidis* MC58 uptake by hCMEC/D3 until 6h but not 8h infection (Fig 4H), possibly due to increasing expression of *S1PR2* (Fig 4C). Therefore, the contribution of S1PR2 to *N*. *meningitidis* uptake by hCMEC/D3 was confirmed by siRNA knockdown of *S1PR2* that reduced expression of *S1PR2* by 80% without affecting expression of *S1PR1* or *S1PR3* (S2B Fig). Downregulation of S1PR2 significantly reduced numbers of recovered intracellular *N*. *meningitidis* MC58 even 8h p.i. (Fig 4I). In support of these findings, pre-treatment with selective S1PR2 agonist CYM5520 [46] significantly increased *N*. *meningitidis* uptake 4h p.i. (Fig 4J), indicating that S1PR2 activation directly precedes and enhances *N*. *meningitidis* endocytosis. Taken together, our results suggest that an increased release of S1P, together with enhanced expression of S1PR2 triggered by *N*. *meningitidis*, leads to transactivation of EGFR, which allows the pathogen to invade BECs.

### Targeting S1PR1 and S1PR3 interferes with *N*. *meningitidis* invasion into BECs

**Fig 5:**
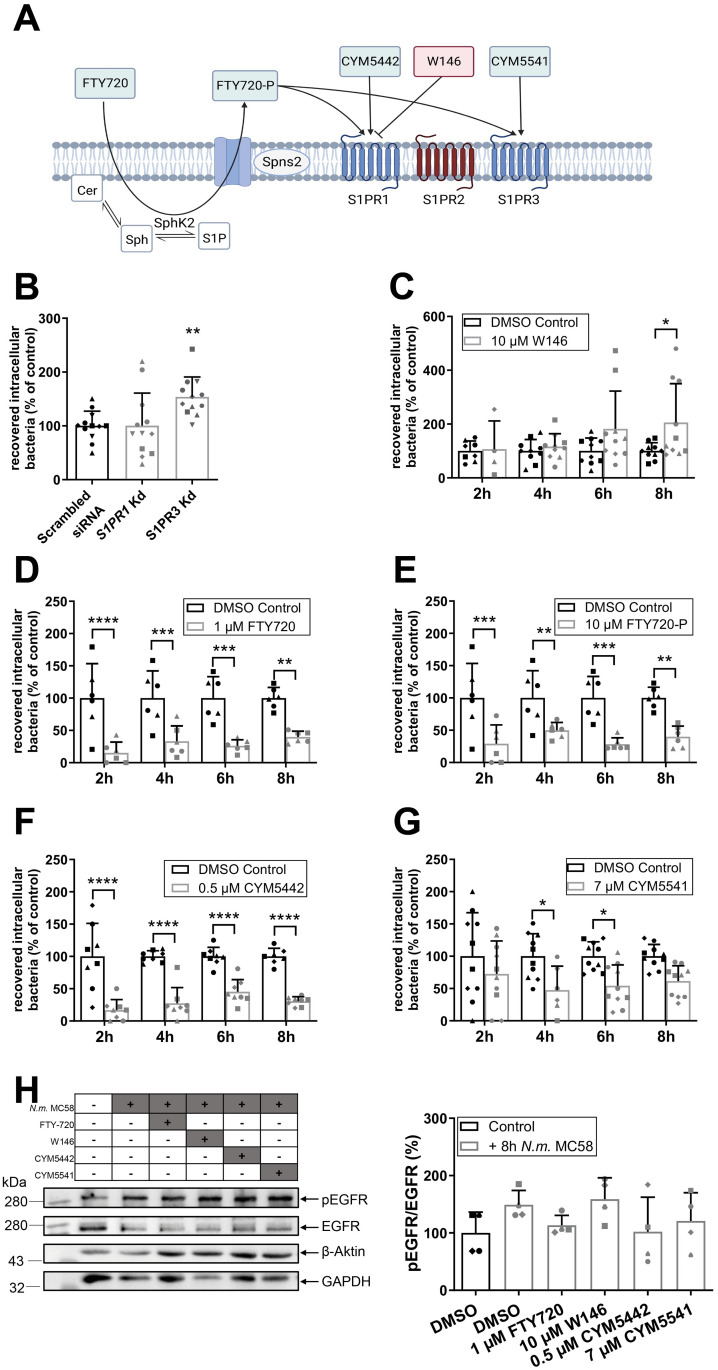
Targeting S1PR1 or S1PR3 by either RNAi-mediated silencing or selective agonists/antagonist treatment interferes with bacterial uptake. (A) Schematic outline of S1PR1 and S1PR3 specific agonists/antagonists used in this study. (B) hCMEC/D3 were transfected with *S1PR1-siRNA (*25 nM), *S1PR3-siRNA* (50 nM) or scrambled siRNA (50 nM) for 72h and infected with *N*. *meningitidis* MC58 for 8h. Numbers of viable intracellular bacteria were determined by gentamicin protection assay and normalized to control cells (scrambled siRNA). Data are means ± SD; n = 6, N = 2. One-way ANOVA followed by Dunnett’s post-hoc test, ** p < 0.01. Absence of cytotoxicity and knockdown efficiency were verified as shown in S2 Fig. (C-G) hCMEC/D3 were pre-treated with S1PR antagonist W146 (C), S1PR1+3–5 agonist FTY-720 (D), S1PR1+3–5 agonist FTY-720-P (E), S1PR1 agonist CYM5442 (F) or S1PR3 agonist CYM5541 (G) or solvent control (DMSO) for 30 min and were subsequently infected with *N*. *meningitidis* MC58 for 8h. Numbers of viable intracellular bacteria were determined by gentamicin protection assay and normalized to control cells (DMSO treated cells). Data are means ± SD; n ≥ 3, N = 2. Multiple t-test and p-value adjustment with Holm-Sidak correction. * p < 0.05, ** p < 0.01, *** p < 0.001, **** p < 0.0001. (H) Assessment of pharmacological modulation of S1PR1 and S1PR3 on EGFR protein levels and phosphorylation at Tyr-845. hCMEC/D3s were pretreated with FTY720, W146, CYM5442 or CYM5541 or solvent control (DMSO) and infected with *N*. *meningitidis* MC58 or left uninfected for 8h. Cell lysates were analyzed by Western blot and subsequent densitometric analysis. Data represent means ± SD, n = 4. One-way ANOVA followed by Dunnett’s post-hoc test showed no significance. Absence of cytotoxicity and bacteriostatic effects were verified as shown in S5 Fig.

Although expression of *S1PR1* and *S1PR3* was not altered during the 8h infection time course (Fig 4B and 4D), both receptors may be involved in the interaction of *N*. *meningitidis* with BECs after the release of S1P. Targeted siRNA knockdown decreased mRNA expression of S1PR1 by 77% (S2A Fig) and mRNA expression of S1PR3 by 71% (S2C Fig), respectively. Transient knockdown of *S1PR3* surprisingly led to an increase in intracellular bacteria, whereas knockdown of *S1PR1* had no effect on *N*. *meningitidis* invasion into BECs (Fig 5B).

To further investigate the contribution of S1PR1/3 to bacterial uptake, we then examined the effects of various S1PR1/3 receptor modulators (summarized in Fig 5A). All antagonist/agonists were used at concentrations, that were not cytotoxic and did not affect bacterial growth (S5 Fig). hCMEC/D3s were treated with the solvent alone or with 10 μM of S1PR1-specific antagonist W146 [47] for 30 min prior to infection with *N*. *meningitidis* MC58. In contrast to the results from the *S1PR1* knockdown experiments, pharmacological targeting of S1PR1 using W146 resulted in an increased number of intracellular bacteria (Fig 5C), suggesting that signaling via S1PR1 and S1PR3 tend to prevent bacterial uptake. To further corroborate this finding, we assessed the effect of FTY-720, a non-selective agonist for 4 of the 5 known S1P receptors, S1PR1,3–5. Treatment of hCMEC/D3s with FTY-720 resulted in significant reduction of bacterial uptake (Fig 5D).

FTY-720 is phosphorylated by sphingosine kinase 2 (SphK2) to function as a S1P receptor ligand [48], but was also found to inhibit SphK1 [49]. To confirm that the reduction in bacterial invasion caused by FTY-720 was due to interaction with the receptor rather than interference with intracellular signaling from SphKs, we next examined the effects of the phosphorylated form of FTY-720, FTY-720-P. As observed for the treatment of hCMEC/D3s with FTY-720, pre-treatment with FTY-720-P resulted in significant reduction of bacterial uptake (Fig 5E).

To assess the individual role of S1PR1 CYM5442, a highly selective S1PR1 agonist, was tested [50]. Treatment of hCMEC/D3s with CYM5442 significantly reduced the number of intracellular *N*. *meningitidis* throughout the course of infection (Fig 5F). We also tested the S1PR3-specific agonist CYM5541 to identify the contribution of S1PR3 [51]. S1PR3-specific agonist CYM5541 also reduced the number of intracellular *N*. *meningitidis* (Fig 5G) although this effect was not as pronounced as with the S1PR1 agonist.

To determine whether S1PR1/3-coupled G proteins transactivate EGFR, hCMEC/D3s were pre-treated with the agonists or antagonists used above, then infected with *N. meningitidis* for 8h and cell lysates were analyzed for p(Tyr845)-EGFR phosphorylation. Even though S1PR1+3 agonists showed a tendency to reduce EGFR phosphorylation, analyses of Western blots did not show significant effects upon S1PR1/3 treatment (Fig 5H). Taken together our results suggest that activation of S1PR1 or S1PR3 signaling prevents *N*. *meningitidis* invasion into BECs. However, this effect cannot be explained by the prevention of EGFR phosphorylation, hence indicating that S1PR1+3 signaling reduces *N*. *meningitidis* uptake by a different mechanism.

### SphKs control *N*. *meningitidis* invasion into BECs

**Fig 6:**
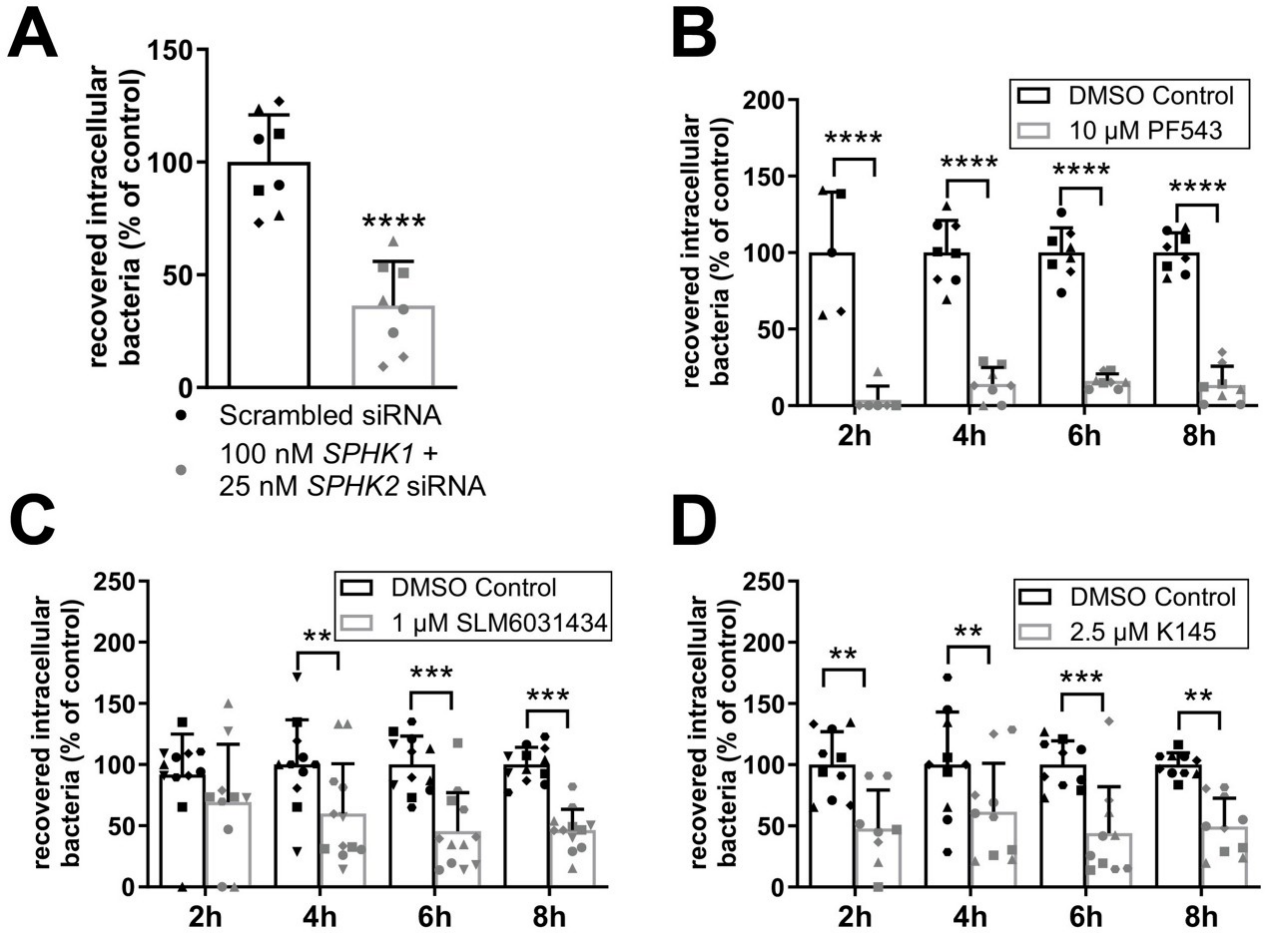
Targeting SphKs by either RNAi-mediated silencing or selective inhibitors reduces bacterial uptake. (A) hCMEC/D3 were co-transfected with *SPHK1-siRNA (100* nM) and *SPHK2-siRNA* (25 nM) or transfected with scrambled siRNA (50 nM) for 24h and infected with *N*. *meningitidis* MC58 for 8h. Numbers of viable intracellular bacteria were determined by gentamicin protection assay and normalized to control cells (scrambled siRNA). Data are means ± SD; n = 4, N = 2. Unpaired students t-test, **** p < 0.0001. Absence of cytotoxicity and knockdown efficiency were verified as shown in S3 Fig. (B-D) hCMEC/D3 were treated with SphK1 inhibitor PF543 (B), SphK2 inhibitors SLM6031434 (C) or SphK2 inhibitor K145 (D) for 30 min or solvent control (DMSO) and subsequently infected with *N*. *meningitidis* MC58 for 8h. Numbers of viable intracellular bacteria were determined by gentamicin protection assay and normalized to control cells (DMSO treated cells). Data are means ± SD; n ≥ 4, N = 2. Comparison to DMSO-treated control using unpaired students t-test (A), multiple t-test with p-value adjustment with Holm-Sidak correction in comparison to control for each time point (B-D). ** p < 0.01, *** p < 0.001, **** p < 0.0001. Absence of cytotoxicity and bacteriostatic effects were verified as shown in S6 Fig.

Having shown the ability of *N*. *meningitidis* to activate SphK in brain endothelial cells and that the resulting increase in S1P release triggers bacterial uptake through the activation of S1PR2 signaling, we wanted to confirm the specific role of SphKs in controlling *N*. *meningitidis* infection. *SPHK1* expression was reduced by more than 57% after treatment with siRNA targeting *SPHK1*, however it also caused significant upregulation of *SPHK2* (S3A Fig). Similar, siRNA targeting SphK2 resulted in reduced *SPHK2* expression by more than 70% and upregulation of highly expressed *SPHK1* (S3B Fig). We therefore set out to simultaneously knock down both isoforms. Double knockdown of both SphKs resulted in reduced levels of *SPHK1* mRNA by more than 70% and *SPHK2* mRNA by more than 80%, respectively (S3C Fig). Remarkably, the double knockdown of *SPHK1* and *SPHK2* expression resulted in a significant inhibition of bacterial uptake at 8h p.i. (Fig 6A). To further determine which isoform is involved in interference with bacterial uptake we used several pharmacological inhibitors that specifically either inhibit SphK1 or SphK2. All inhibitor concentrations tested had no cytotoxic effects on hCMEC/D3 or had any effect on bacterial growth (S6 Fig). Treatment of hCMEC/D3s with PF543, a potent and specific inhibitor of SphK1 [52], greatly reduced the number of intracellular *N*. *meningitidis* over an 8h infection time course (Fig 6B). We next explored the effect of hCMEC/D3 treatment using two highly selective and potent SphK2 inhibitors, SLM6031434 [53] or K145 [54] (Fig 6C and 6D). While treatment with the inhibitors did not affect bacterial adherence (S6 Fig), the numbers of intracellular *N*. *meningitidis* significantly lowered, although this effect was not as pronounced as with SphK1 inhibition.

## Disscussion

In this study we report a significant increase of S1P in BECs infected with *N*. *meningitidis* that correlated with increased SphK1 expression and activity. Increased SphK activity was found to be dependent on Tfp interaction with its cognate receptor CD147. S1P in turn was exported out of cells and activated S1PR2, whose expression was increased during infection. S1PR2 signaling activated EGFR and thus regulated bacterial uptake. In agreement, inhibiting SphK or S1PR2 activation markedly reduced *N*. *meningitidis* invasion into BECs. Collectively, our findings disclose a crucial role of S1P and S1PR2 in *N*. *meningitidis* invasion and establish a novel signaling mechanism, resembling criss-cross transactivation, induced by the pathogen, leading to SphK1 activation, mediating signals between three individual ligand-receptor systems (Tfp/CD147, S1P/S1PR2 and EGFR).

Previous work from our group reports that the pathogen also transiently activates the enzyme ASMase, that hydrolyses sphingomyelin to ceramide and phosphocholine, thus increasing levels of ceramides on the plasma membrane of BECs [6, 7]. Surface ceramides form large ceramide-enriched membrane platforms (CRPs) that serve to reorganize and cluster receptor molecules and *N*. *meningitidis* uses such CRPs as entry portal [6, 7, 55]. It is noteworthy that the activation of the enzyme ASMase can be attributed to the expression of Tfp [7], as we also showed here in this study for SphK activity. Moreover, SphK activation could also be prevented by ASMase inhibition. Two endothelial cell receptors are important for Tfp, CD147, a member of the immunoglobulin (Ig) superfamily, and the β2-adrenergic receptor [31, 32, 56]. *N*. *meningitidis* first interacts with the membrane proximal IgG domain of CD147. Here we delineated that the interaction between Tfp and the membrane proximal IgG domain of CD147 is necessary for SphK activation.

The use of LC-MS/MS measurements taken at different time points allowed us to establish a time-resolved profile of the sphingolipid pathway and thereby allowed us to quantify the formation of ceramides during *N*. *meningitidis* infection of BECs. Moreover, we observed a significant time-dependent increase of cellular sphingosine and S1P, peaking 2h and 4h p.i., respectively. The transient elevation in sphingosine and S1P induced by *N*. *meningitidis* suggests that ceramide is subsequently degraded by ceramidase to produce sphingosine, the substrate for SphK. Sphingosine may also play a role in bacterial infection. Of note, dihydroceramides were also elevated in the cellular compartment as well as in the supernatants. Moreover, further studies are needed to determine how S1P, ceramides or dihydroceramides are released from hCMEC/D3s and whether hCMEC/D3s release extracellular vesicles upon *N*. *meningitidis* infection.

Importantly, *N*. *meningitidis* induced increased expression of S1PR2 in BECs, suggesting a shift in the S1PR expression pattern on hCMEC/D3 cells after meningococcal infection towards S1PR2-mediated signaling that is crucial for barrier disruption. Blocking of S1PR2 signaling resulted in impaired EGFR phosphorylation at Tyr-845 and abrogated bacterial uptake. EGFR can be activated downstream of G protein-coupled receptors (GPCRs) by two different mechanisms. The mechanisms involved in GPCR-induced EGFR signaling can occur through either the intracellular or extracellular pathways [57]. Intracellularly, G proteins transactivate EGFR via Src-mediated phosphorylation at Tyr-845; extracellularly, released EGFR-like ligands stimulate EGFR directly. We previously reported that *N*. *meningitidis* induce Src-mediated phosphorylation of EGFR at Tyr-845 and this step is involved in the uptake process [35]. Furthermore, we found that the EGFR is activated by the release of the ligand HB-EGF in response to *N*. *meningitidis* and depletion of HB-EGF results in reduced numbers of invasive bacteria [35], indicating that both pathways are relevant to *N*. *meningitidis* invasion into BECs. Here, we provided evidence that demonstrates the capacity of S1P to stimulate EGFR transactivation through the S1PR2 receptor in BECs infected with *N*. *meningitidis*. By blocking S1PR2 signaling, the highly selective S1PR2 antagonist JTE-013 attenuated EGFR phosphorylation at Tyr-845, indicating that transactivation of EGFR is mediated by Src signaling.

EGFR is considered an important receptor manipulated by diverse pathogens for their survival in the host [58–60]. It was recently shown that transactivated EGFR recruits α-actinin-4, an actin-cross-linking protein, from intracellular F-actin fibers, thereby disrupting the cytoskeleton to facilitate *E*. *coli* invasion of BECs [61]. Of note, α-actinin-4 recruitment is also relevant for *N*. *meningitidis* adhesion, as α-actinin-4 binds directly to the cytosolic tail of CD147 and directs the assembly of CD147-β2AR complexes in highly ordered clusters at bacterial adhesion sites [32]. Whether α-actinin-4 recruitment may also play a role in S1PR2-EGFR-mediated uptake of *N*. *meningitidis* requires further investigation.

S1PR2 signaling via Rho/ROCK can induce vascular permeability through stress fiber formation and disruption of adherence junctions. ROCK can directly destabilize tight junctions by phosphorylation of tight junction proteins occludin and claudin-5 [62]. In addition, ROCK-mediated phosphorylation of the myosin-binding subunit of myosin-light chain phosphatase (MCLP) inhibits MCLP activity and hence leads to cellular contraction through reduced MCL dephosphorylation [63]. Moreover, ROCK can phosphorylate MCL and therefore directly contributes to cellular contraction [64]. Overexpression of S1PR2 in HUVECs completely abolishes S1P-induced cortical actin formation, whereas S1PR2 inhibition with JTE-013 prior to S1P treatment enhances cortical actin formation, indicating a fine-tuned regulation of cortical actin stabilization through different S1P receptors [65]. It should be mentioned that S1PR2 is the only S1PR that can activate both the small GTPase Rho and ERM (ezrin/radixin/moesin), a family of adaptor molecules linking the cortical actin cytoskeleton to the plasma membrane [66]. This might be relevant to its unique action as it has been suggested that pilus-mediated adhesion of *N*. *meningitidis* recruit ERM and together with Rho are critical for the subsequent cytoskeletal modifications responsible for the formation of protrusions and bacterial internalization [67].

Surprisingly, targeting S1PR3 by RNAi-mediated silencing or targeting S1PR1 with selective antagonist W146 increased bacterial uptake into BECs. No effect on bacterial uptake was however observed after treatment with S1PR1 siRNA, possibly due to a low turn-over rate or remaining expression of S1PR1. Treatment with S1PR1-specific agonist CYM5442 as well as S1PR3-specific agonist CYM5541, however, resulted in reduced bacterial uptake suggesting that binding of S1P to these receptors reduces numbers of invasive bacteria. Here, we showed that EGFR phosphorylation was only slightly affected by modulation of S1PR1/3 signaling, suggesting that these receptors influence bacterial entry via a different mechanism. Interestingly, both S1PR1 and S1PR3 increase the stability of the endothelial barrier by strengthening the cortical ring through Rac-dependent mechanisms. S1P exerts its barrier-strengthening effect via Rac-dependent translocation of cortactin to the cell periphery, but also requires the interaction of cortactin with endothelial myosin light chain kinase MLCK [68]. While S1P signaling via S1PR1 enhances α-actinin 1/4 recruitment via Rac/PI3K/Tiam1 [69], activation S1PR1+3 leads to the inactivation of cofilin via Rac/PAK/LIMK resulting in reduced severing and overall stabilization of the of the cortical actin network [70]. Conversely, constant actin disassembly by cofilin is essential for the formation of membrane ruffles, which form the phagocytic cup as a pre-requisite for cellular uptake as shown for example for *Listeria monocytogenes* [71]. It is conceivable that *N*. *meningitidis* entry into hCMEC/D3s was inhibited by a similar mechanism, and future studies will address the question of whether activation of S1PR1+3 stabilizes the cortical cytoskeleton of BECs and inhibits the formation of membrane protrusions to prevent *N*. *meningitidis* uptake.

Several pathogenic bacteria can bind to and cross the endothelial cells of the BBB and cause meningitis [72, 73]. Consistent with our study, meningitic *E*. *coli* have also been shown to modulate sphingolipid homeostasis in BECs [74]. In contrast to *N*. *meningitidis*, meningitic *E*. *coli* induce phosphorylation of SphK2 releasing S1P, thus resulting in transactivation of EGFR downstream of S1PR2 important for *E*. *coli* penetration of the BBB [74].

S1P receptors are variably coupled to three heterotrimeric G proteins, G_αi_, G_12/13_ and G_q_. While S1PR1 couples exclusively to the G protein G_αi_, S1PR2/3 couple to all three G proteins, G_αi_, G_12/13_ and G_q_ [75]. *In vitro* studies indicate that S1PR1-G_αi_ coupling directly acts on Rac, leading to rearrangement of the cytoskeleton of endothelial cells and formation of a cortical ring, resulting in cellular spreading and flattening [70]. In addition, by binding to S1PR1, S1P increases translocation of the adherence junction component VE-cadherin to cell-cell contact regions to further stabilize endothelial barrier integrity [76]. Opposingly, S1PR2 via coupling to G_12/13_, leads to endothelial leakage through Rho-mediated Rac inhibition [65]. The descripted function of S1PR3 is more controversial. While S1PR3 knockdown mice are protected against LPS-induced disruption of the lung endothelial barrier [77], *in vitro* experiments show that S1PR3 along-side with S1PR1 promotes endothelial barrier function via G_αi_ signaling [70].

The functional balance between signal transduction after activation of S1PR1/3 and S1PR2, respectively, at the BBB is well established, and confers to protection of barrier integrity via regulation of tight junction proteins or cytoskeletal rearrangement [22]. While plasma S1P levels are controlled systemically, it is possible that S1P levels at the BBB are controlled through local changes of S1P modulating enzymes and therefore S1P receptor signaling at the BBB could be controlled by endothelial cells in an autocrine manner by S1P release and S1P receptor expression [78, 79]. Future studies are needed to determine the impact of S1P and S1PRs on the BBB disruption in response to *N*. *meningitidis* infection.

Despite advancements in antibacterial therapy, bacterial meningitis remains one of the most important infectious diseases worldwide [80]. The most common pathogens in children and adults include *N*. *meningitidis*, which is associated with high morbidity and mortality. Here we show that *N*. *meningitidis* modulates the sphingolipid content on BECs to efficiently invade BECs. Our study provides new insights into the underlying mechanisms—which involves SphK1 activation and a novel signaling mechanism, that mediates signals between three individual ligand-receptor system—and thus a basis for the development of a potential adjuvant strategy to combat bacterial meningitis caused by this pathogen.

## Materials and methods

### Cell line and culture conditions

hCMEC/D3 cells (derived from human temporal lobe microvessels isolated from tissue removed during operations for control of epilepsy [81], Merck) were cultured in EndoGRO-MV Complete Culture Medium (Merck) supplemented with 20 ng/L hBFGF (Merck) on 150 μg/mL collagen 1 (Gibco) coated T75 flasks at 37°C and 5% CO_2_. For sub-cultivation and infection experiments, hCMEC/D3 were detached using Trypsin/EDTA solution (ThermoFisher) and seeded in optimum density. Cells from passage 10 to 25 were used for experiments. hCMEC/D3 cell line was authenticated by ATCC cell line authentication service using short tandem repeat analysis [82]. Cells were regularly checked for mycoplasma contaminations using a commercially available kit (PanReac AppliChem).

### Bacterial strains and growth conditions

*Neisseria meningitidis* serogroup B strain MC58 (sequence type [ST74]/ST-32 clonal complex (CC)) [83] is a clinical isolate from UK and was kindly provided by E.R. Moxon. *N*. *meningitidis* strain 8013/clone 12 (also designated 2C4.3) is a piliated capsulated variant of the serogroup C meningococcal clinical isolate 8013 ([ST77]/ST8 CC) and was kindly provided by M. Taha [84]. Generation of nonpiliated mutant *N*. *meningitidis* 8013 *pilE* was reported in a previous study [7]. *N*. *meningitidis* wildtype strains were grown on Columbia sheep blood agar plates (bioMérieux) and incubated overnight at 37°C at 5% CO_2_. Mutant strains were grown on GC agar plates supplemented with spectinomycin (75 μg/mL, Merck). Liquid cultures were prepared by transferring bacteria from agar plates to proteose pepton medium (Merck) supplemented with 1% Kelloggs reagent [85], 10 nM NaHCO_3_ (Merck) and 10 nM MgCl_2_ (Carl Roth) (PPM+) and additionally supplemented with spectinomycin (75 μg/mL) for isogenic mutants. Liquid cultures were incubated with shaking (200 rpm) for 90 min at 37°C.

To generate an isogenic non piliated mutant of strain MC58, the *pilE* gene was amplified using oligonucleotides *pilE*_*Xba*I_fwd and *pilE*_*Xho*I_rev, cleaved with *Xba*I and *Xho*I, respectively, and cloned into the pTL1 vector [86]. The pTL1 plasmid, harboring the *pilE* gene fragment was then transformed into TOP 10 chemically competent *E*. *coli* cells. After confirmation of positive clones by nucleotide sequencing, an inverse PCR was performed using olidonucleotides *pilE*_*Avr*II_inv_fwd and *pilE*_*Avr*II_inv_rev harboring an *Avr*II restriction site, the resulting construct was cutted with *Avr*II and a spectinomycin-resistance cassette was inserted via the *Avr*II sites by replacing about 400 bp of the coding sequence. The resultant construct was amplified in *E*. *coli* TOP10 before transformation of the plasmid into *N*. *meningitidis* strain MC58. *N*. *meningitidis* MC58 *pilT* mutant, which lacks the pilus retraction machinery, was obtained with the same cloning strategy using *pilT*_*Xba*I_fwd, *pilT*_*Xho*I_rev, *pilT*_*Avr*II_inv_fwd and *pilT*_*Avr*II_inv_rev, respectively. All primer sequences are listed in Table 1. Mutant strains were confirmed by PCR, sequencing, and Western blot analysis.

**Table 1:** Oligonucleotides used in this study.

Oligonuclaotide	Sequence (with restriction site)
*pilT*_*Xba*I_fwd	gcgcgcTCTAGAACAAAGCATCCGACCTTCAC
*pilT*_*Xho*I_rev	gcgcgcCTCGAGACCAGCGATTGCAGCGATT
*pilT*_*Avr*II_inv_fwd	TGGTGCCCTAGGTGCTGTCCGAATCGCTGAC
*pilT*_*Avr*II_inv_rev	TGCGGACCTAGGCGCGTTGGCGAAGCTGAG
*pilE*_*Xba*I_fwd	gcgcgcTCTAGAGTACCAACAAGGCTGGATTC
*pilE*_*Xho*I_rev	gcgcgcCTCGAGCGTTGCCTCGGCTTAGCTC
*pilE*_*Avr*II_inv_fwd	GTAAAACCTAGGTGCGGACAGCCGGTTACG
*pilE*_*Avr*II_inv_rev	TGTTGCCCTAGGATTCGCCGTGATTCAGG

### Inhibitors, agonists, and antagonists

All Inhibitors, agonists and antagonists were purchased from commercial sources and dissolved in DMSO (Carl Roth) (final DMSO concentration of 0.1%). For infection experiments, PF543 (Echelon Biosciences), FTY-720-phosphate (Echelon Biosciences), W146 (Merck) and JTE-013 (Merck) were dissolved in DMSO to yield a stock solution of 10 mM. FTY-720 (Echelon Biosciences), CYM5520 (Cayman Chemicals) and SLM-6031434 (Tocris) were dissolved in DMSO to yield a stock solution of 1 mM. K145 (Merck) was dissolved to a stock solution of 2.5 mM, CYM5442 (Cayman Chemicals) to a stock solution of 0.5 mM and CYM5541 (Cayman Chemicals) to a stock solution of 7 mM, all in DMSO.

### Cell viability assay

The cytotoxic effects of all inhibitors, agonists, antagonists, or siRNA against SphKs used in this study were determined via flow cytometric measurement of propidium iodide positive cells. In brief, hCMEC/D3s were seeded on 24-well plates, grown until confluence and treated with inhibitors, agonists, antagonists, or solvent as negative control for 8.5 h. For cytotoxicity measurements of RNAi experiments, cells were transfected with indicated siRNA concentrations or scrambled siRNA for indicated periods of time and incubated in EndoGRO medium supplemented with hBFGF for 8.5 h. Cells were washed with PBS, trypsinized for 5 min and centrifuged at 1500 g for 5 min. Cells were stained for 10 min on ice with 150 μL propidium iodide (10 μg/mL, Merck) / ribonuclease A (RNAse A) (2.5 mg/mL, Merck) solution and afterwards diluted by adding 150 μL of PBS. hCMEC/D3s treated with 0.25% Triton X-100 (Carl Roth) served as positive control. Flow cytometric measurement of at least 10,000 gated events per sample was conducted using MACS Quant10 (Miltenyi). The percentage of propidium iodide negative cells was determined from 10,000 events measured after exclusion of cellular debris and doublets via forward and sideward scatter using FlowJo analysis software.

### Growth curves

Bacteria from liquid cultures were centrifuged at 3320 x g for 5 min, resuspended in cell culture medium and optical density at 600 nm (OD_600_) was measured to calculate the bacterial inoculum (OD_600_ = 1 ≙ 1*10^9 bacteria/mL). 1*10^8 bacteria were diluted in 10 mL EndoGRO medium supplemented with hBFGF containing indicated concentrations of inhibitors, agonist, or antagonist (S5 and S6 Figs) or DMSO as negative control and incubated with shaking (200 rpm) at 37°C. OD_600_ were recorded every 30 min for 180 min.

### Infection assays

Bacteria were seeded a day before the infection assay on Columbia sheep blood agar plates or GC agar plates supplemented with spectinomycin from a stock conserved at -80°C. Bacteria were transferred to 10 mL of PPM+ for subsequent growth under agitation for 90 min. Bacteria from liquid cultures were centrifuged at 3320 xg for 5 min, resuspended in cell culture medium and CFUs were evaluated by OD_600_ measurements and diluted to provide a multiplicity of infection (MOI) of 100. hCMEC/D3 cells were seeded onto different well plate sizes coated with 150 μg/mL collagen 1 and grown until confluence. EndoGRO medium supplemented with hBFGF was replaced 24h prior to infection with serum-starvation medium (sterile-filtered EndoGRO Basal medium (Merck) + 0.1% BSA (PanReac AppliChem)) to synchronize the cells. On the day of infection, cells were washed once with PBS, and EndoGRO medium supplemented with hBFGF containing the respective inhibitors, agonists, or antagonists or 0.1% DMSO as negative control was added 30 min prior to infection. Gentamicin protection assays were then performed as previously described [6]. Numbers of adherent and invasive bacteria were normalized to the controls of the individual experiments.

### LC-MS/MS measurement

hCMEC/D3 cells were grown until confluency in 6-well plates, reaching approximately 1.5*10^6 cells/well at the day of infection, and were infected as described using an MOI of 100. At indicated time points, supernatants (1 mL) were collected and mixed with 3 mL 1-butanol (Carl Roth) for lipid extraction as described [87]. In parallel, cells were washed with PBS, trypsinized for 5 min at 37°C and collected in a reaction tube. After centrifugation for 5 min at 2000 g, supernatant was discarded and cells were resuspended in 1.5 mL methanol/chloroform (2:1, v:v) (Carl Roth/Supelco) for lipid extraction as described [88]. For work-up of both, supernatants and cells, the extraction solvent contained the internal standards d_7_-sphingosine (d_7_-Sph), d_7_-sphingosine 1-phosphate (d_7_-S1P), 17:0 Cer and d_31_-16:0 SM (all Avanti Polar Lipids). Chromatographic separations were achieved on a 1290 Infinity II HPLC (Agilent Technologies) equipped with a Poroshell 120 EC-C8 column (3.0 × 150 mm, 2.7 μm; Agilent Technologies). MS/MS analyses were carried out using a 6495C triple-quadrupole mass spectrometer (Agilent Technologies) operating in the positive electrospray ionization mode (ESI+). HPLC conditions and ion source settings of the MS/MS detector have been described elsewhere [89]. Sphingolipid subspecies were quantified by multiple reaction monitoring (MRM) using the following mass transitions (qualifier product ions in parentheses): long-chain bases: *m/z* 300.3 → 282.3 (252.3) for Sph, *m/z* 307.3 → 289.3 (259.3) for d_7_-Sph, *m/z* 380.3 → 264.3 (82.1) for S1P, and *m/z* 387.3 → 271.3 (82.1) for d_7_-S1P; ceramides: *m/z* 520.5 → 264.3 (282.3) for 16:0 Cer, *m/z* 534.5 → 264.3 (282.3) for 17:0 Cer, *m/z* 548.5 → 264.3 (282.3) for 18:0 Cer, *m/z* 576.6 → 264.3 (282.3) for 20:0 Cer, *m/z* 604.6 → 264.3 (282.3) for 22:0 Cer, *m/z* 630.6 → 264.3 (282.3) for 24:1 Cer, and *m/z* 632.6 → 264.3 (282.3) for 24:0 Cer; sphingomyelins: *m/z* 703.6 → 184.1 (86.1) for 16:0 SM, *m/z* 731.6 → 184.1 (86.1) for 18:0 SM, *m/z* 734.6→ 184.1 (86.1) for d_31_-16:0 SM, *m/z* 759.6 → 184.1 (86.1) for 20:0 SM, *m/z* 787.7 → 184.1 (86.1) for 22:0 SM, *m/z* 813.7 → 184.1 (86.1) for 24:1 SM, and *m/z* 815.7 → 184.1 (86.1) for 24:0 SM. Peak areas of Cer and SM subspecies, as determined with MassHunter Quantitative Analysis software (version 10.1, Agilent Technologies), were normalized to those of the internal standards (17:0 Cer or d_31_-16:0 SM) followed by external calibration in the range of 1 fmol to 50 pmol on column. Sph and S1P were directly quantified via their deuterated internal standards d_7_-Sph (0.25 pmol on column) and d_7_-S1P (0.125 pmol on column). Changes in sphingolipid content were calculated by normalizing the determined concentrations to the concentrations of uninfected cells for each experiment and for S1P to the respective time point.

### RT-qPCR

hCMEC/D3s were infected as described above. Sample collection and RNA purification was performed according to NucleoSpin RNA purification protocol (Marcherey Nagel). In brief, 350 μL RA1 buffer containing 1% 2-Mercaptoethanol (Merck) were added, technical duplicates were pooled to ensure sufficient RNA yield. and frozen until RNA purification was carried out. RNA concentration and purity was measured with NanoDrop One (ThermoFisher). 500 ng RNA were used for cDNA synthesis with LunaScript RT SuperMix (New England BioLabs) according to manufacturer’s protocol. cDNA samples were diluted 1:10 in nuclease-free H_2_O and used for RT-qPCR with PowerUp SYBR Green master mix (ThermoFisher) and 200 nM of respective primers listed in Table 2. RT-qPCR was performed using StepOnePlus real-time PCR thermocycler (ThermoFisher) and analyzed using StepOnePlus software (ThermoFisher). Fold change gene expression compared to negative control was calculated in Microsoft Excel following the delta delta Ct method with *18S* rRNA as reference gene.

**Table 2:** Primers used for qPCR.

Gene	Forward sequence	Reverse sequence
*18S rRNA* ^a^	GTAACCCGTTGAACCCCATT	CCATCCAATCGGTAGTAGCG
*SPHK1* ^b^	CCTTCACGCTGATGCTCACT	GCAATAGCGTGCAGTTGGTC
*SPHK2* ^c^	AGCGTGGTAGCCACTTCAG	GAGCAGTGTACCGATGCCA
*SGPP1* ^d^	CCATTTGTGGACCTGATTGACA	ACTTCCTAGTATCTCGGCTGTG
*SGPP2* ^d^	TCACCGCACTCCTCATCGT	CCGGGTTGGGCTGTAGTAATC
*SGPL1* ^e^	ACGAAGATGATGGAGGTGGATG	TGAGGAAACTGTGGGGTAGAAC
*SPNS2* ^d^	GACAGCAGAGACGTGCAACA	CCAGAAATCCCGTAAAGCAGG
*S1PR1*	CTCCGTGTTCAGTCTCCTCG	ATTGCTCCCGTTGTGGAGTT
*S1PR2* ^f^	CATCGTCATCCTCTGTTG	AGTGGAACTTGCTGTTTC
*S1PR3* ^d^	CGGCATCGCTTACAAGGTCAAC	GCCACGAACATACTGCCCTC
*S1PR4*	CTGTATGGGGAGCAGGGAAC	AGGGTGCTCTCTGCTCCTAC
*S1PR5* ^d^	GCGCACCTGTCCTGTACTC	GTTGGTGAGCGTGTAGATGATG
*CXCL1* ^g^	CTCTTCCGCTCCTCTCACAG	GGGGACTTCACGTTCACACT
*CXCL2* ^g^	CTCAAGAATGGGCAGAAAGC	AAACACATTAGGCGCAATCC
*CCL20* ^g^	GCGCAAATCCAAAACAGACT	CAAGTCCAGTGAGGCACAAA
*IL6* ^g^	GGAGACTTGCCTGGTGAAAA	CAGGGGTGGTTATTGCATCT
*IL8* ^h^	AGCTCTGTGTGAAGGTGCAG	AATTTCTGTGTTGGCGCAGT

^a^Rho et al 2010 [90].

^b^Forward primer from Suh et al 2018 [91].

^c^Dong et al 2012 [92].

^d^Havard Primer Bank [93].

^e^Spassieva et al 2012 [94].

^f^Jeya et al [79].

^g^van Sorge et al [95].

^h^Kim et al [96].

For all Primers used for RT-qPCR, annealing temperature was optimized and efficiency (E = 95–105%) and linearity (pearson R > 0.999) was validated. Specificity was controlled by melt curve analysis and agarose gel electrophoresis.

### Preparation of pilus-enriched fractions

Pilus-enriched fractions (PeF) were prepared as previously described [7]. Briefly, 50 plates of *N*. *meningitidis 8013*/12 or *N*. *meningitidis* 8013/12 *pilE* were incubated overnight. The next day, the total bacterial content was swapped and transferred to 60 mL ethanolamine solution (150 mM, pH = 10.5) in PBS. The solution was vortexed for 2 min to shear off pilus proteins. Bacterial cells and smaller debris were removed by a first centrifugation step at 12,000 x g for 10 min followed by a second centrifugation step at 21,500 x g for 90 min. The supernatant was then transferred to an Erlenmeyer flask and 6.6 mL ammonium sulfate-saturated (0.7 g/mL, Merck) ethanolamine solution (150 mM in PBS, pH = 10.5, Carl Roth) was added and stirred for 30 min on a magnetic mixer. The resulting precipitate, containing the pilus proteins, was pelleted through centrifugation at 21,000 x g for 15 min. The supernatant was discarded, and the pellets were dissolved in 50 mL TBS (0.05 M TRIS, pH = 7.5, Carl Roth). The protein solution was transferred to a VivaSpin column (50 mL, molecular weight cut-off 5 kDa, Merck). The columns were centrifuged at 4000 x g until the volume of the upper fraction was reduced to 1 mL. Protein content was determined using Pierce BCA assay (ThermoFisher). Pilus-enrichment and absence of Opc was verified by Western blotting. 1 μg of purified proteins (PeF) or TBS as solvent control was used for treatment of cells.

### Sphingosine kinase assay

Activity of sphingosine kinases was determined using a commercially available sphingosine kinase activity assay kit (Echelon Biosciences # K-3500). In brief, hCMEC/D3s were grown on 96-well plates, pre-treated and infected as described. At indicated time points, cells were washed once with PBS before adding 50 μL of the reaction buffer containing 0.1% dithiothreitol (DTT). Cell lysis was achieved by three repeated freeze-thawn cycles. 10 μL of cell lysate was mixed with 400 μM sphingosine (in 10 μL reaction buffer containing 0.1% DTT) and 20 μM ATP (in 20 μL containing 0.1% DTT) in 96-well plates. The plates were sealed and incubated for 2 h at 37°C. After incubation, 40 μL of luciferase-based ATP detector was added for further 10 min to stop the kinase reaction. Kinase activity was tested using SpectraMax iD3 ELISA reader (Molecular Devices). To test the specificity of the commercially available ATP depletion-based assay, we validated that baseline SphK activity was significantly reduced without addition of Sph as substrate (Fig 2H) as well as after siRNA-based knockdown of SphK1 and SphK2 (S1A Fig).

For blocking the interaction between Tfp and the receptor CD147, 10 μg/mL of anti-CD147 (clone MEM-M6/6, antibody, that binds to the extracellular N-terminal domain, Biorad # MCA2882Z) or 10 μg/mL anti-CD147 (clone MEM-M6/1, antibody, that binds to the C-terminal Ig domains, Biorad # MCA1876) were used and added 30 min before treatment of cells with PeF.

### Western blot analysis

For determination of EGFR protein level and phosphorylation, hCMEC/D3s were grown on 6-well plates in conditioned medium and left either uninfected, infected with *N*. *meningitidis* or treated with S1P or EGF as positive controls. S1P (Avanti Polar Lipids) was dissolved in methanol: water according to the manufacturer’s protocol and diluted to the desired concentration of 125 μM in fat-free BSA (4 mg/mL in PBS). At indicated time points, supernatants were removed, cells were washed with 500 μL ice-cold PBS and harvested in 300 μL ice-cold modified RIPA2 buffer (50 mM TRIS-HCl (pH = 7.2), 150 mM NaCl (Carl Roth), 5 mM EDTA (PanReac), 1% Triton X-100, 24.1 mM Sodium deoxycholate (Merck), 0.1% SDS (Carl Roth), 50 mM Sodium fluoride (Merck), 1% protease and phosphatase inhibitor cocktail (ThermoFisher)). Cells were collected using a cell scraper, agitated on a rotary shaker for 45 min on ice and lysates were centrifuged at 12,000 x g for 20 min at 4°C. Lysates were diluted in 2x SDS reducing sample buffer (140 mM TRIS-HCl (pH 6.8), 22.4% Glycerol (Carl Roth), 4.5% SDS, 6.9% 2-Mercaptoethanol, Bromophenol blue (Merck)). For the determination of EGFR expression and phosphorylation, lysates were incubated for 10 min at 95°C.

For determination of SphK1/2 expression and phosphorylation, cell lysates were generated using the same protocol, however lysates were incubated for 10 min at 70°C. Cytosolic and plasma membrane fractions were isolated using a plasma membrane protein isolation kit (Invent BioTechnologies). In brief, hCMEC/D3s from two 175cm^2^ flasks (appr. 5*10^7^ cells) were scraped in ice-cold PBS and treated according to the manufacturer’s protocol without the filtration step to prevent nucleic burst. Total protein content of cytosolic and plasma membrane fractions was measured by Pierce BCA Assay (ThermoFisher). Equal protein amounts were loaded on SDS Gels.

Samples were probed on 12% SDS-PAGE gels and transferred to nitrocellulose membranes (Merck). Then, membranes were blocked with 5% BSA in TBS-T 1 h at RT and probed with phospho-specific antibodies at 4°C o/n, washed three times with TBS-T. Anti-rabbit or anti-mouse secondary HPR-Antibody (JacksonResearch Labs) were used to incubate the membranes for 1h at RT and membranes were washed 3 times with TBS-T. Finally, the protein bands were detected with ECL substrate (Bio-Rad) and imaged using a chemiluminescence detector (Bio-Rad). Blots were subsequently stripped with mild stripping buffer (200 mM Glycine (Carl Roth), 0.1% SDS, 1% Tween20, pH = 2.2) and then reprobed with anti-SphK1 (Cell Signalling), anti-SphK2 (Proteintech), or anti-EGFR (Santa Cruz), washed and incubated with HRP-conjugated secondary antibody. Primary and secondary antibodies with recommended dilution are given in Table 3. GAPDH and β-Actin (Proteintech) served as protein loading controls. The blot images were quantified using FIJI ImageJ analysis software, and the values for each phosphorylated target protein were normalized to target protein expression. The values for phosphorylation levels in infected cells were calculated relative to the values of phosphorylation levels in uninfected control cells.

**Table 3:** Antibodies used in this study.

Primary Antibody	Dilution	diluent
Rabbit monoclonal Anti-SphK1, Clone D1H1L (Cell Signalling # 12071S)	1:1000	5% Skim milk in TBS-T
Rabbit polyclonal Anti-SphK2 (Proteintech, # 17096-1-AP)	1:1000	5% Skim milk + 1% BSA in TBS-T
Rabbit polyclonal pospho-specific Anti-(Ser-225)-SphK1(ECM biosciences # SP1641)	1:250	5% BSA in TBS-T
Rabbit polyclonal pospho-specific Anti-(Thr-578)-SphK2 pospho-specific (ECM biosciences # SP4631)	1:500	5% BSA in TBS-T
Mouse monoclonal Anti-EGFR (Santa Cruz Biotechnology # sc-373746)	1.1000	5% Skim milk in TBS-T
Mouse monoclonal pospho-specific Anti-(Thr-845)-EGFR, clone 12A3 (Santa Cruz # sc-57542)	1:200	5% Skim milk in TBS-T
Rabbit monoclonal pospho-specific Anti-(Thr-845)-EGFR, clone D63B4 (Cell signaling # 6963)	1:1000	5% Skim milk in TBS-T
Mouse monoclonal Anti-GAPDH, clone 1E6D9 (Proteintech # 60004-1-Ig)	1:20000	5% BSA in TBS-T
Mouse monoclonal Anti-β-Actin, clone 2D4H5 (Proteintech # 66009-1-Ig)	1:20000	5% Skim milk in TBS-T
**Secondary antibodies**		
Goat polyclonal Anti-Rabbit IgG, F(ab’)2 Fragment—Horseradish Peroxidase conjugate (JacksonResearch Labs # 111-035-006)	1:5000	in respective diluent of primary antibody
Goat polyclonal Anti-Mouse IgG + IgM (H+L)—Horseradish Peroxidase conjugate (JacksonResearch Labs # 115-035-044)	1:10000	in respective diluent of primary antibody

### siRNA transfection

hCMEC/D3s were transfected using TransIT-siQUEST (MIRUS) transfection reagent following the manufacturer’s protocol. In brief, 2*10^5 cells per well were seeded in 24-well plates in 500 μL EndoGRO medium supplemented with hBFGF. The next day, 1.5 μL of TransIT-siQUEST transfection reagent and indicated amounts of siRNA were added to 50 μL EndoGRO Basal medium (Merck) and incubated for 30 min. The transfection mix was added dropwise to cells and incubated for indicated time points, but at least 24 h for efficient transfection. Cells were transfected with siRNA specific for SPHK1 (Santa Cruz # sc-44114), SPHK2 (Santa Curz # sc-39225), EDG-1 (S1PR1) (Santa Cruz # sc-37086), EDG-5 (S1PR2) (Santa Cruz # sc-39928) or EDG-3 (S1PR3) (Santa Cruz # sc-35261). FITC-labeled scrambled siRNA (Santa Cruz # sc-36869) was used as negative control and transfection efficiency was controlled by fluorescence microscopy using an Eclipse Ti-E inverted microscope (Nikon). Cells were subsequently serum-starved for 24 h prior to experiments as described. Efficiency of knockdown was recorded using RT-qPCR. Cytotoxic effects were measured using propidium iodide staining.

### Statistics

Data represent mean ± standard deviation. Statistical significances of at least 3 independent experiments were calculated using GraphPad Prism version 6.01 (GraphPad Software Inc.). Pairwise comparisons were carried out using two-tailed students t-test. If multiple t-tests were employed, p-values were adjusted with Holm-Sidak correction. Comparisons with more than two groups were carried out using one- or two-way ANOVA followed by Dunnett’s post-hoc test to determine significance compared to control group. P values lower than 0.05 were considered significant. Experiment replicates and statistical analyses are indicated in the figure legends. Information about data normalization can be found in the figure legends.

### Software

Calculations were performed and graphs were created in Microsoft Excel version 2303 and GraphPad Prism version 6.01. Western Blot band intensity quantification was done using FIJI ImageJ version 1.52 [97]. LC-MS/MS raw data were analyzed with MassHunter Quantitative Analysis 10.1 (Agilent Technolgies). Figs were prepared using Adobe Illustrator Version 27.5. Figs 2A, 3C and 5A and the graphical abstract were created using biorender.com.

## Supporting information

S1 Fig Validation of SphK Assay specificity and determination of SphK1 recruitment to the plasma membrane. (A) SphK enzymatic activity measured after 24h knockdown using 100 nM SPHK1+ 25 nM SPHK2 siRNA or scrambled siRNA. hCMEC/D3s were infected with *N*. *meningitidis* MC58 for 4h or left uninfected prior to lysis and measurement. Data are presented as means ± SD, n = 4. Two-way ANOVA followed by Dunnett’s post-hoc test for comparison to scrambled siRNA control. ***p<0.001,****p<0.0001. (B) Western blot detection of SphK1 phopshorylation in cytosolic and plasma membrane fractions after 4h infection with *N*. *meningitidis* MC58. Equal amounts of protein were loaded and stained for SphK1 as well as SphK1 (pSer-225).
(TIF)

S2 Fig Determination of S1PR1, S1PR2, and S1PR3 knockdown their specific siRNAs and cytotoxic effects. (A-C) mRNA expression of *S1PR1*, *S1PR2* and *S1PR3* after knockdown of (A) S1PR1, (B) S1PR2 or (C) S1PR3. Expression fold change relative to *18S* rRNA was determined by qRT-PCR after 72h transfection with siRNA specific for S1PR1, S1PR2, or S1PR3 (12.5, 25 and 50 nM), 24h starvation with serum-free EndoGRO and 8.5h incubation in full medium. Data represent mean ± SD, n = 3. One-way ANOVAs followed by Dunnett’s post-hoc test for comparison to 50 nM scrambled siRNA control were performed to identify target or off-target effects. *p<0.05, **p<0.01, *** p < 0.001. (D) Cytotoxicity of transfection with 50 nM siRNA specific for *S1PR1* or *S1PR3* was determined for hCMEC/D3 using propidium iodide staining followed by flow-cytometric analysis. hCMEC/D3s treated with 50 nM scrambled siRNA were used as negative control. 0.25% Triton-X100 served as positive control. Data represent mean ± SD, n = 3. One-way ANOVAs followed by Dunnett’s post-hoc test for comparison to scrambled siRNA control. ****p<0.0001.
(TIF)

S3 Fig Determination of SPHK1 or SPHK2 knockdown by their specific siRNAs and cytotoxic effects. (A+B) mRNA expression of (A) *SPHK1* and (B) *SPHK2* after knockdown of target gene or the respective other *SPHK*. Expression fold change relative to *18S* rRNA was determined by qRT-PCR after 24h transfection with siRNA specific for *SPHK1* or *SPHK2* (25, 50, 100 nM), 24h starvation with serum-free EndoGRO and 8.5h incubation in full medium. Data represent mean ± SD, n = 3. One-way ANOVAs followed by Dunnett’s post-hoc test for comparison to 100 nM scrambled siRNA control were performed to identify target or off-target effects. ** p < 0.01. (C) mRNA expression of *SPHK1* and *SPHK2* after double knockdown of *SPHK1 and SPHK2*. Expression fold change relative to *18S* rRNA was determined by qRT-PCR after 24h co-transfection with siRNA specific for *SPHK1* and *SPHK2*, 24h starvation with serum-free EndoGRO and 8.5h incubation in full medium. Data represent mean ± SD, n = 3. One-way ANOVAs followed by Dunnett’s post-hoc test for comparison to target gene expression of 100 nM scrambled siRNA control. ** p < 0.01, *** p < 0.001. (D) Cytotoxicity of co-transfection with *SPHK1* and *SPHK2* siRNA was determined for hCMEC/D3 using propidium iodide staining and flow-cytometric analyses. hCMEC/D3s treated with 100 nM scrambled siRNA were used as negative control. 0.25% Triton-X100 served as positive control. Data represent mean ± SD. One-way ANOVA followed by Dunnett’s post-hoc test for comparison to scrambled siRNA control. n = 3, *p<0.05, ****p<0.0001.
(TIF)

S4 Fig Determination of cytotoxic and bacteriostatic effects of JTE-013 and CYM5520. (A+D) Inhibitory effects on bacterial growth of *N*. *meningitidis* MC58 were determined by optical density (OD) measurement of *N*. *meningitidis* liquid culture in full medium for 180 min time course after addition of (A) JTE-013, (D) CYM5520 or DMSO as solvent control. Data represent mean ± SD, n = 3. Two-way ANOVA followed by Dunnett’s post-hoc test for comparison to DMSO control showed no significant effects. (B+E) Cytotoxicity of 8.5h treatment with (A) JTE-013 and (B) CYM5520 was determined with propidium iodide staining and flow-cytometric analyses. hCMEC/D3s were treated with respective concentrations of S1PR2 modulators or DMSO as negative control. 0.25% Triton-X100 served as positive control. Data represent mean ± SD. n = 3. One-way ANOVA followed by Dunnett’s post-hoc test for comparison to DMSO control. ****p<0.0001. (C+F) The effects of (C) JTE-013 or (F) CYM5520 on the adherence of *N*. *meningitidis* MC58 to hCMEC/D3s over an 8h time course were determined using gentamicin protection assays. CFU counts were normalized to individual control. Data represent mean ± SD. n ≥ 4 in duplicates. Comparison to DMSO-treated control for each time point using multiple t-test with p-value adjustment with Holm-Sidak, *p<0.05.
(TIF)

S5 Fig Determination of cytotoxic and bacteriostatic effects of W146, FTY-720, FTY-720-P, CYM5442 and CYM5541. (A,D,G,J,M) Inhibitory effects on bacterial growth of *N*. *meningitidis* MC58 were determined by OD measurement of *N*. *meningitidis* liquid culture in full medium for 180 min time course in the presence of (A) W146, (D) FTY720, (G) FTY720-phosphate, (J) CYM5442, (M) CYM5541 or DMSO as solvent control. Data represent mean ± SD, n ≥ 3. Two-way ANOVA followed by Dunnett’s post-hoc test for comparison to DMSO control. **p<0.01, ****p<0.0001. (B,E,H,K,N) Cytotoxicity of 8.5h treatment with (B) W146, (E) FTY720, (H) FTY720-phosphate, (K) CYM5442 or (N) CYM5541 was determined with propidium iodide staining and flow-cytometric analyses. hCMEC/D3s were treated with respective concentrations of S1PR1/3 modulators or DMSO as control. 0.25% Triton-X100 served as positive control. Data represent mean ± SD. n ≥ 3. One-way ANOVA followed by Dunnett’s post-hoc test for comparison to DMSO negative control. ***p<0.0001, ****p<0.0001. (C,F,I,L,O) The Effects of (C) W146, (F) FTY720, (I) FTY720-phosphate, (L) CYM5442 or (O) CYM5541 on the adherence of *N*.*meningitidis* MC58 to hCMEC/D3s over an 8h time course were determined using gentamicin protection assays. CFU counts were normalized to individual control. Data represent mean ± SD. n ≥ 4 in duplicates. Comparison to DMSO-treated control for each time point using multiple t-test with p-value adjustment with Holm-Sidak correction, *p<0.05.
(TIF)

S6 Fig Determination of cytotoxic and bacteriostatic effects of PF543, SLM6031434 and K145. (A,D,G) Inhibitory effects on bacterial growth of *N*. *meningitidis* MC58 was determined by OD measurement of *N*. *meningitidis* liquid culture in full medium for 180 min time course in the presence of (A) PF543, (D) SLM6031434, (G) K145 or DMSO as solvent control. Data represent mean ± SD, n ≥ 3 in duplicate. Two-way ANOVA followed by Dunnett’s post-hoc test for comparison to DMSO control. *p<0.05, **p<0.01, ****p<0.0001. (B,E,H) Cytotoxicity of 8.5h treatment with (B) PF543, (E) SLM6031434 or (H) K145 was determined with propidium iodide staining and flow-cytometric analyses. hCMEC/D3s were treated with respective concentrations of SphK inhibitors or DMSO as negative control. 0.25% Triton-X100 served as positive control. Data represent mean ± SD, n = 3, N = 2. One-way ANOVA followed by Dunnett’s post-hoc test for comparison to DMSO control. ***p<0.0001, ****p<0.0001. (C,F,I) The effects of (C) PF543, (F) SLM6031434, or (I) K145 on the adherence of *N*. *meningitidis* MC58 to hCMEC/D3s over an 8h time course were determined using gentamicin protection assays. CFU counts were normalized to individual control. Data represent mean ± SD. n ≥ 3. Comparison to DMSO-treated control for each time point using multiple t-test with p-value adjustment with Holm-Sidak correction showed no significant changes.
(TIF)

S1 Table List of Reagents and Materials used in this study. (DOCX)
