# Supplementary figures and images for: Sphingosine kinase 1/S1P receptor signaling axis is essential for cellular uptake of Neisseria meningitidis in brain endothelial cells
Source: PLoS Pathog. 2023 Nov 30;19(11):e1011842. doi: 10.1371/journal.ppat.1011842 (PMC10715668; doi:10.1371/journal.ppat.1011842)

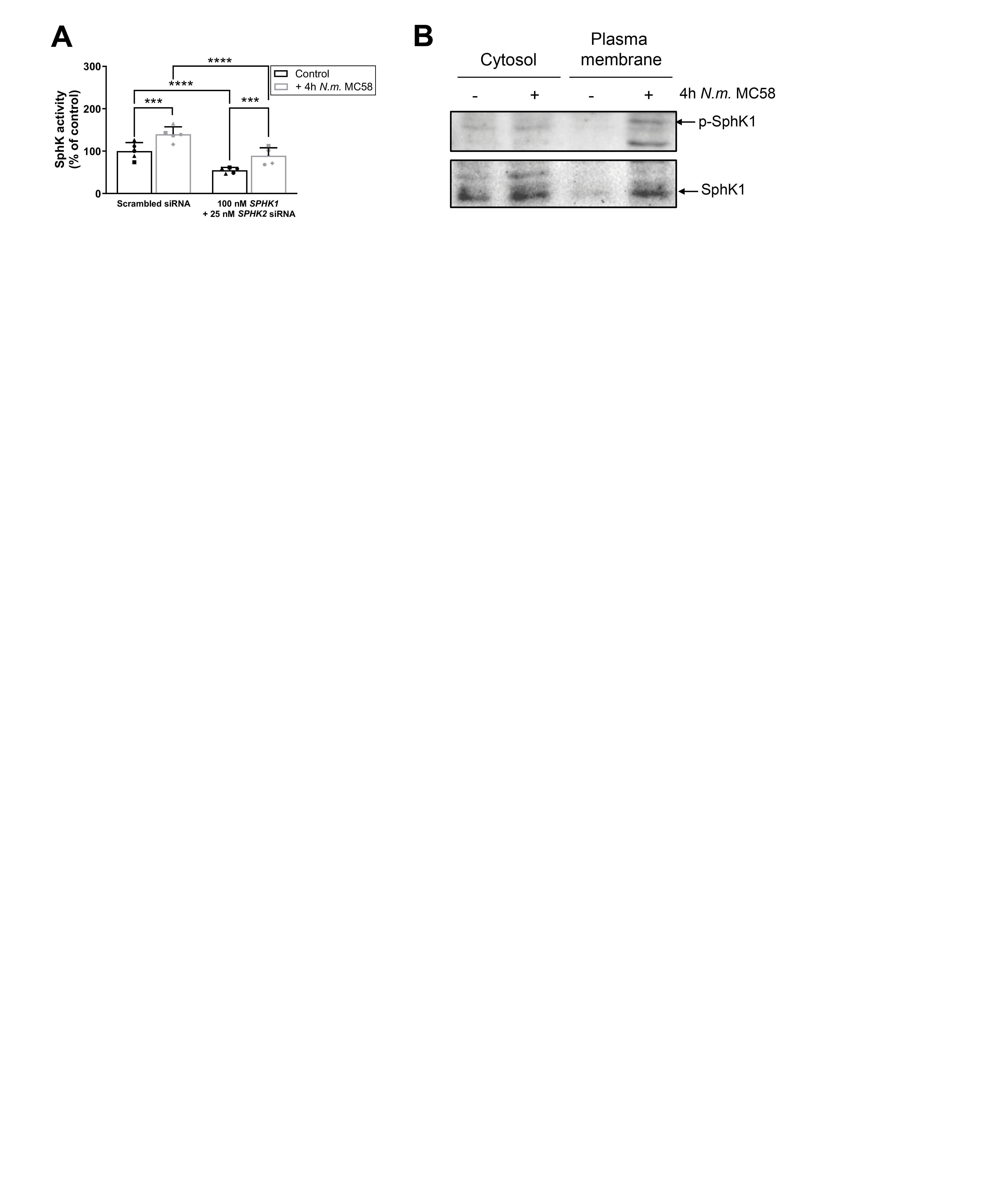

Supplement: S1 Fig — (A) SphK enzymatic activity measured after 24h knockdown using 100 nM SPHK1+ 25 nM SPHK2 siRNA or scrambled siRNA. hCMEC/D3s were infected with N. meningitidis MC58 for 4h or left uninfected prior to lysis and measurement. Data are presented as means ± SD, n = 4. Two-way ANOVA followed by Dunnett’s post-hoc test for comparison to scrambled siRNA control. ***p<0.001,****p<0.0001. (B) Western blot detection of SphK1 phopshorylation in cytosolic and plasma membrane fractions after 4h infection with N. meningitidis MC58. Equal amounts of protein were loaded and stained for SphK1 as well as SphK1 (pSer-225). (TIF) [file ppat.1011842.s001.tif]

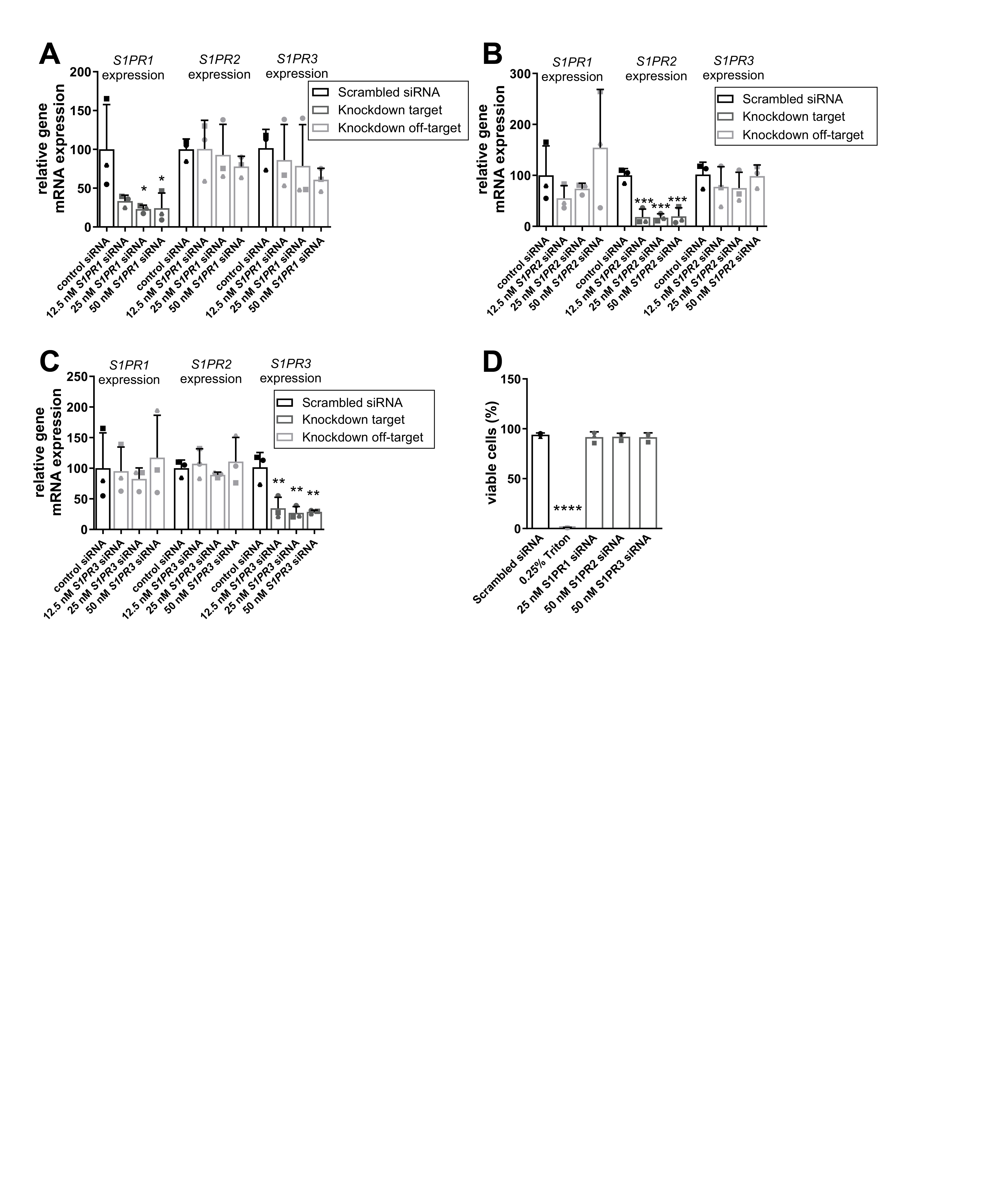

Supplement: S2 Fig — (A-C) mRNA expression of S1PR1, S1PR2 and S1PR3 after knockdown of (A) S1PR1, (B) S1PR2 or (C) S1PR3. Expression fold change relative to 18S rRNA was determined by qRT-PCR after 72h transfection with siRNA specific for S1PR1, S1PR2, or S1PR3 (12.5, 25 and 50 nM), 24h starvation with serum-free EndoGRO and 8.5h incubation in full medium. Data represent mean ± SD, n = 3. One-way ANOVAs followed by Dunnett’s post-hoc test for comparison to 50 nM scrambled siRNA control were performed to identify target or off-target effects. *p<0.05, **p<0.01, *** p < 0.001. (D) Cytotoxicity of transfection with 50 nM siRNA specific for S1PR1 or S1PR3 was determined for hCMEC/D3 using propidium iodide staining followed by flow-cytometric analysis. hCMEC/D3s treated with 50 nM scrambled siRNA were used as negative control. 0.25% Triton-X100 served as positive control. Data represent mean ± SD, n = 3. One-way ANOVAs followed by Dunnett’s post-hoc test for comparison to scrambled siRNA control. ****p<0.0001. (TIF) [file ppat.1011842.s002.tif]

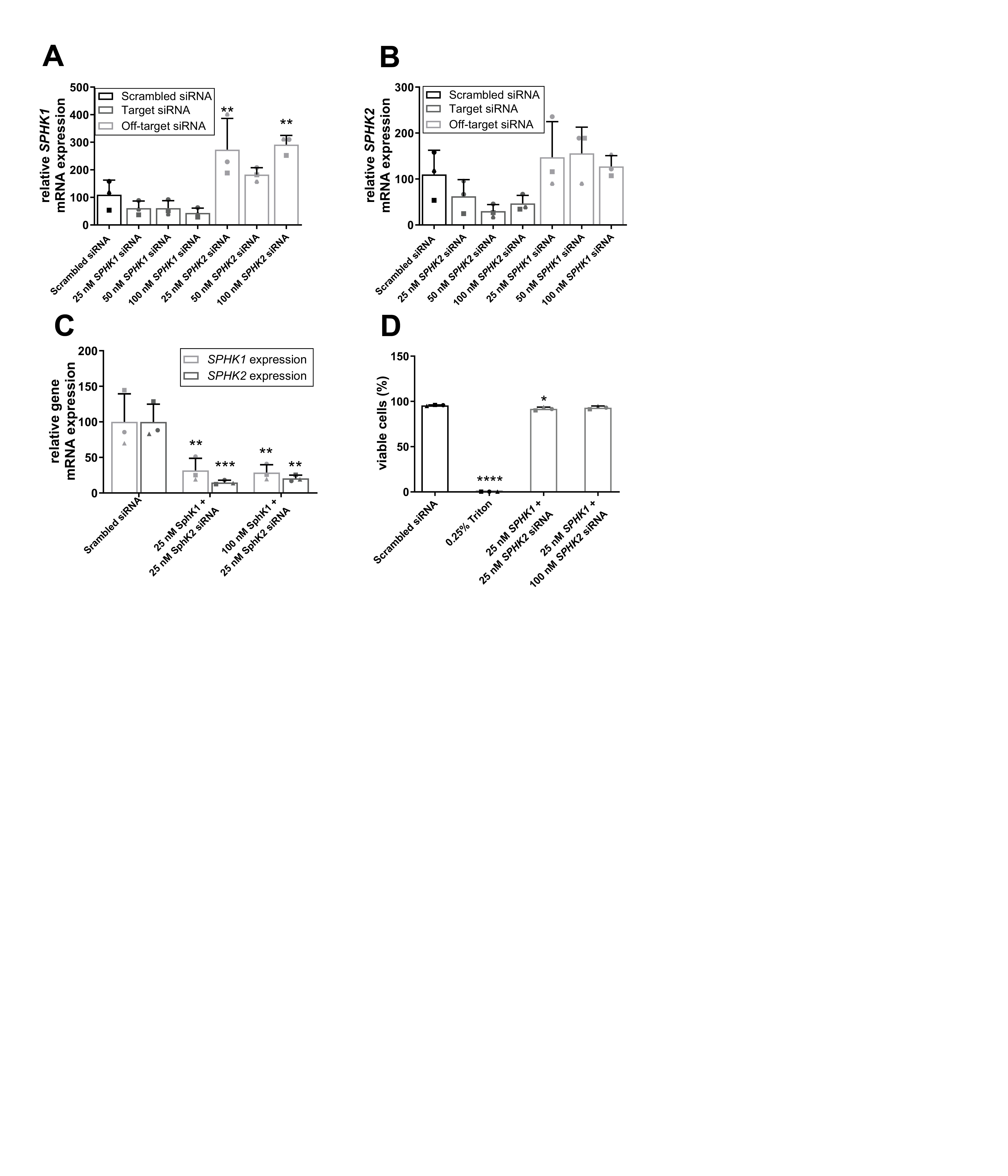

Supplement: S3 Fig — (A+B) mRNA expression of (A) SPHK1 and (B) SPHK2 after knockdown of target gene or the respective other SPHK. Expression fold change relative to 18S rRNA was determined by qRT-PCR after 24h transfection with siRNA specific for SPHK1 or SPHK2 (25, 50, 100 nM), 24h starvation with serum-free EndoGRO and 8.5h incubation in full medium. Data represent mean ± SD, n = 3. One-way ANOVAs followed by Dunnett’s post-hoc test for comparison to 100 nM scrambled siRNA control were performed to identify target or off-target effects. ** p < 0.01. (C) mRNA expression of SPHK1 and SPHK2 after double knockdown of SPHK1 and SPHK2. Expression fold change relative to 18S rRNA was determined by qRT-PCR after 24h co-transfection with siRNA specific for SPHK1 and SPHK2, 24h starvation with serum-free EndoGRO and 8.5h incubation in full medium. Data represent mean ± SD, n = 3. One-way ANOVAs followed by Dunnett’s post-hoc test for comparison to target gene expression of 100 nM scrambled siRNA control. ** p < 0.01, *** p < 0.001. (D) Cytotoxicity of co-transfection with SPHK1 and SPHK2 siRNA was determined for hCMEC/D3 using propidium iodide staining and flow-cytometric analyses. hCMEC/D3s treated with 100 nM scrambled siRNA were used as negative control. 0.25% Triton-X100 served as positive control. Data represent mean ± SD. One-way ANOVA followed by Dunnett’s post-hoc test for comparison to scrambled siRNA control. n = 3, *p<0.05, ****p<0.0001. (TIF) [file ppat.1011842.s003.tif]

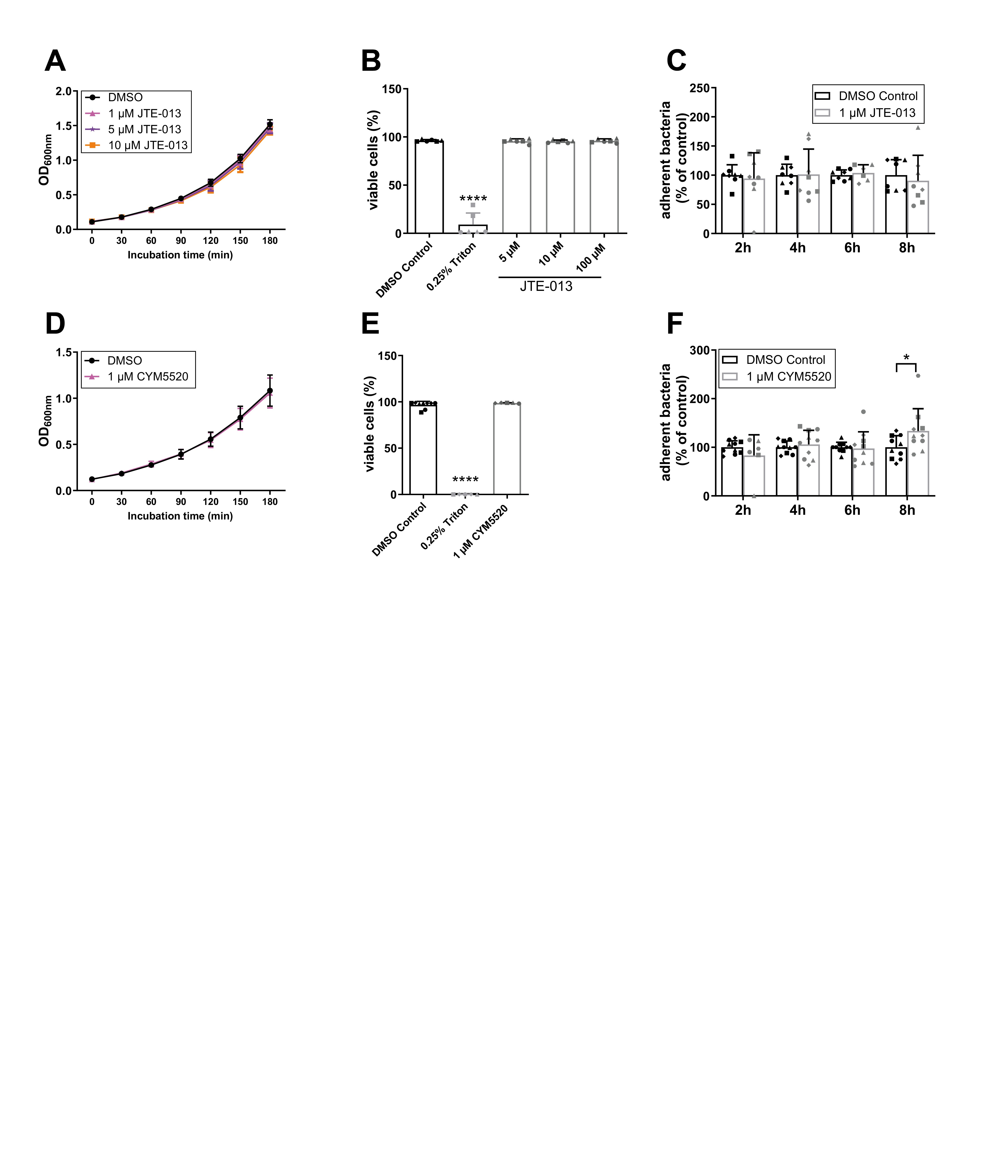

Supplement: S4 Fig — (A+D) Inhibitory effects on bacterial growth of N. meningitidis MC58 were determined by optical density (OD) measurement of N. meningitidis liquid culture in full medium for 180 min time course after addition of (A) JTE-013, (D) CYM5520 or DMSO as solvent control. Data represent mean ± SD, n = 3. Two-way ANOVA followed by Dunnett’s post-hoc test for comparison to DMSO control showed no significant effects. (B+E) Cytotoxicity of 8.5h treatment with (A) JTE-013 and (B) CYM5520 was determined with propidium iodide staining and flow-cytometric analyses. hCMEC/D3s were treated with respective concentrations of S1PR2 modulators or DMSO as negative control. 0.25% Triton-X100 served as positive control. Data represent mean ± SD. n = 3. One-way ANOVA followed by Dunnett’s post-hoc test for comparison to DMSO control. ****p<0.0001. (C+F) The effects of (C) JTE-013 or (F) CYM5520 on the adherence of N. meningitidis MC58 to hCMEC/D3s over an 8h time course were determined using gentamicin protection assays. CFU counts were normalized to individual control. Data represent mean ± SD. n ≥ 4 in duplicates. Comparison to DMSO-treated control for each time point using multiple t-test with p-value adjustment with Holm-Sidak, *p<0.05. (TIF) [file ppat.1011842.s004.tif]

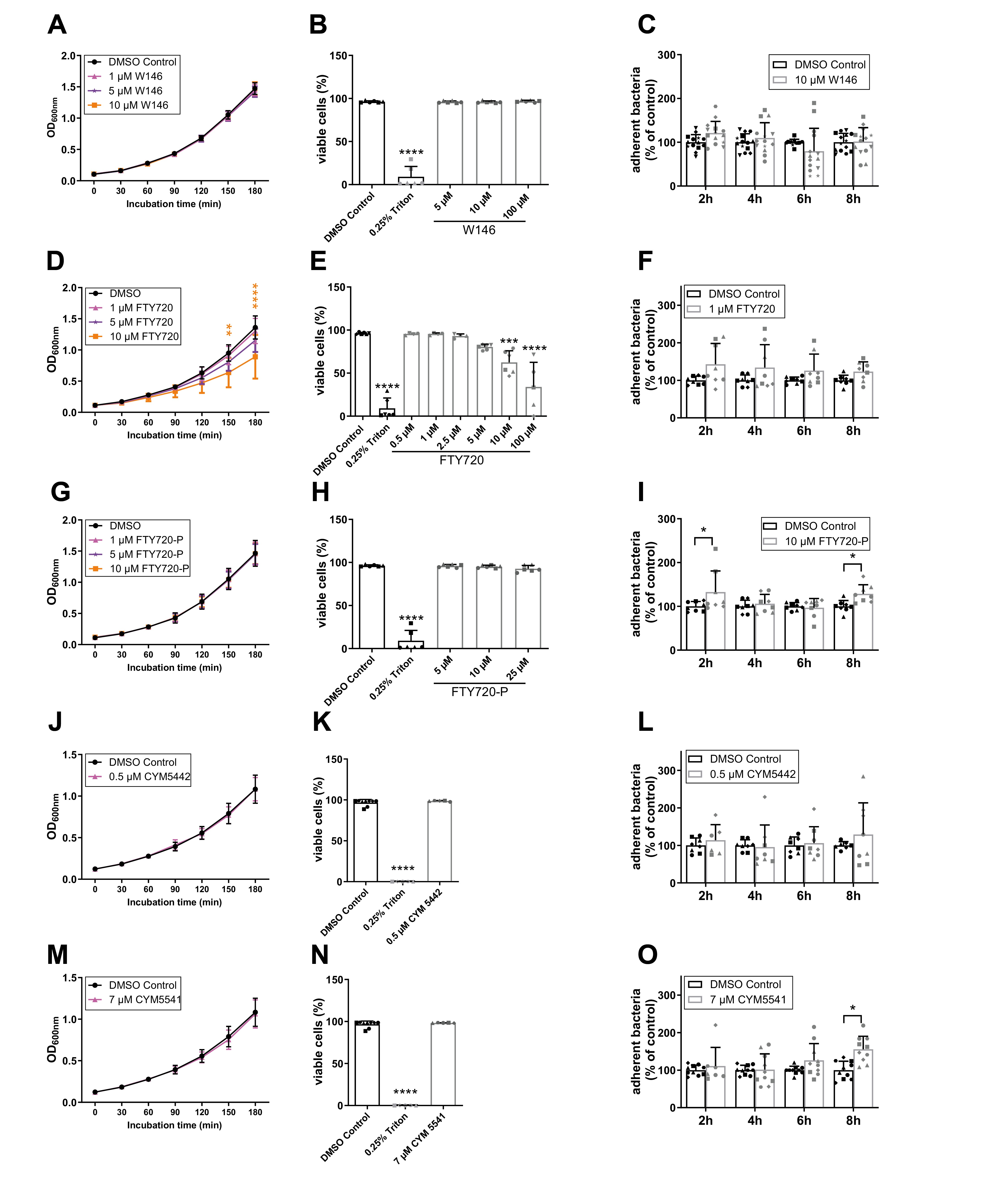

Supplement: S5 Fig — (A,D,G,J,M) Inhibitory effects on bacterial growth of N. meningitidis MC58 were determined by OD measurement of N. meningitidis liquid culture in full medium for 180 min time course in the presence of (A) W146, (D) FTY720, (G) FTY720-phosphate, (J) CYM5442, (M) CYM5541 or DMSO as solvent control. Data represent mean ± SD, n ≥ 3. Two-way ANOVA followed by Dunnett’s post-hoc test for comparison to DMSO control. **p<0.01, ****p<0.0001. (B,E,H,K,N) Cytotoxicity of 8.5h treatment with (B) W146, (E) FTY720, (H) FTY720-phosphate, (K) CYM5442 or (N) CYM5541 was determined with propidium iodide staining and flow-cytometric analyses. hCMEC/D3s were treated with respective concentrations of S1PR1/3 modulators or DMSO as control. 0.25% Triton-X100 served as positive control. Data represent mean ± SD. n ≥ 3. One-way ANOVA followed by Dunnett’s post-hoc test for comparison to DMSO negative control. ***p<0.0001, ****p<0.0001. (C,F,I,L,O) The Effects of (C) W146, (F) FTY720, (I) FTY720-phosphate, (L) CYM5442 or (O) CYM5541 on the adherence of N.meningitidis MC58 to hCMEC/D3s over an 8h time course were determined using gentamicin protection assays. CFU counts were normalized to individual control. Data represent mean ± SD. n ≥ 4 in duplicates. Comparison to DMSO-treated control for each time point using multiple t-test with p-value adjustment with Holm-Sidak correction, *p<0.05. (TIF) [file ppat.1011842.s005.tif]

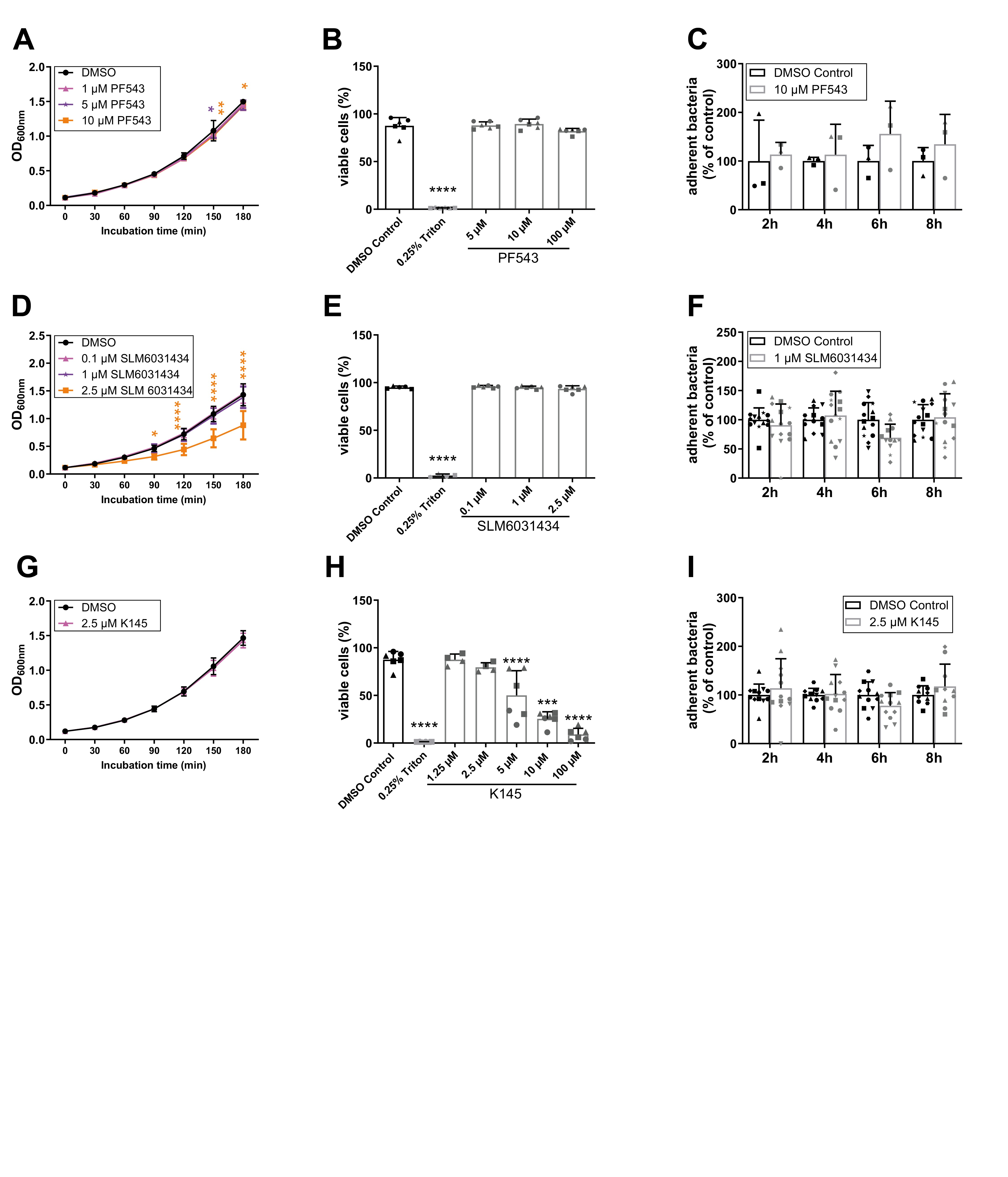

Supplement: S6 Fig — (A,D,G) Inhibitory effects on bacterial growth of N. meningitidis MC58 was determined by OD measurement of N. meningitidis liquid culture in full medium for 180 min time course in the presence of (A) PF543, (D) SLM6031434, (G) K145 or DMSO as solvent control. Data represent mean ± SD, n ≥ 3 in duplicate. Two-way ANOVA followed by Dunnett’s post-hoc test for comparison to DMSO control. *p<0.05, **p<0.01, ****p<0.0001. (B,E,H) Cytotoxicity of 8.5h treatment with (B) PF543, (E) SLM6031434 or (H) K145 was determined with propidium iodide staining and flow-cytometric analyses. hCMEC/D3s were treated with respective concentrations of SphK inhibitors or DMSO as negative control. 0.25% Triton-X100 served as positive control. Data represent mean ± SD, n = 3, N = 2. One-way ANOVA followed by Dunnett’s post-hoc test for comparison to DMSO control. ***p<0.0001, ****p<0.0001. (C,F,I) The effects of (C) PF543, (F) SLM6031434, or (I) K145 on the adherence of N. meningitidis MC58 to hCMEC/D3s over an 8h time course were determined using gentamicin protection assays. CFU counts were normalized to individual control. Data represent mean ± SD. n ≥ 3. Comparison to DMSO-treated control for each time point using multiple t-test with p-value adjustment with Holm-Sidak correction showed no significant changes. (TIF) [file ppat.1011842.s006.tif]
