# Supplementary material for: Sphingosine kinase 1/S1P receptor signaling axis is essential for cellular uptake of Neisseria meningitidis in brain endothelial cells
Source: PLoS Pathog. 2023 Nov 30;19(11):e1011842. doi: 10.1371/journal.ppat.1011842 (PMC10715668; doi:10.1371/journal.ppat.1011842)
Supplement: S1 Table — (DOCX) [file ppat.1011842.s007.docx]

# Reagents and Materials

| **REAGENT or RESOURCE** | **SOURCE** | **IDENTIFIER** |
| --- | --- | --- |
| **Antibodies** | | |
| Mouse monoclonal Anti-CD147, clone MEM-M6/6 | Bio-Rad | Cat# MCA2882Z, RRID:AB_2066685 |
| Mouse monoclonal Anti-CD147, clone MEM-M6/1 | Bio-Rad | Cat# MCA1876, RRID:AB_322225 |
| Goat polyclonal Anti-Rabbit IgG, F(ab')2 Fragment - Horseradish Peroxidase conjugate | Jackson ImmunoResearch Labs | Cat# 111-035-006, RRID:AB_2337936 |
| Goat polyclonal Anti-Mouse IgG + IgM (H+L) - Horseradish Peroxidase conjugate | Jackson ImmunoResearch Labs | Cat# 115-035-044, RRID:AB_2338503 |
| Rabbit monoclonal Anti-SphK1, Clone D1H1L | Cell Signalling | Cat# 12071S, RRID:  AB_2797815 |
| Rabbit polyclonal Anti-SphK2 | Proteintech | Cat# 17096-1-AP, RRID: AB_10598479 |
| Rabbit polyclonal pospho-specific Anti-(Ser-225)-SphK1 | ECM biosciences | Cat# SP1641, RRID: AB_2195826 |
| Rabbit polyclonal pospho-specific Anti-(Thr-578)-SphK2 pospho-specific | ECM biosciences | Cat# SP4631, RRID: AB_10839407 |
| Mouse monoclonal Anti-Opc, clone mAb 306 | Provided by M. Virji |  |
| Mouse monoclonal Anti-SM1, mAb SM1 | Provided by M. Virji |  |
| Mouse monoclonal Anti-EGFR | Santa Cruz Biotechnology | Cat# sc-373746, RRID:AB_10920395 |
| Mouse monoclonal pospho-specific Anti-(Thr-845)-EGFR, clone 12A3 | Santa Cruz Biotechnology | Cat# sc-57542, RRID:AB_783187 |
| Rabbit monoclonal pospho-specific Anti-(Thr-845)-EGFR, clone D63B4 (Cell signaling # 6963) | Cell Signalling | Cat# 6963; |
| Mouse monoclonal Anti-GAPDH, clone 1E6D9 | Proteintech | Cat# 60004-1-Ig, RRID:AB_2107436 |
| Mouse monoclonal Anti-β-Actin, clone 2D4H5 | Proteintech | Cat# 66009-1-Ig, RRID:AB_2687938 |
|  |  |  |
| Bacterial strains | | |
| *Neisseria meningitidis* MC58 | Provided by E.R. Moxon [[1](#_ENREF_1)] |  |
| *Neisseria meningitidis* MC58 *pilT* | this study |  |
| *Neisseria meningitidis* MC58 *pilE* | this study |  |
| *Neisseria meningitidis* 8013/clone 12 | Provided by M. Taha [[2](#_ENREF_2)] |  |
| *Neisseria meningitidis* 8013/clone 12 *pilE* | Peters *et al*. [[3](#_ENREF_3)] |  |
| **Chemicals, peptides, and recombinant proteins** | | |
| EndoGRO-MV Complete Culture Media Kit | Merck | Cat# SCME004 |
| Recombinant Human FGF-basic (bFGF) | ThermoFisher | Cat# 100-18B |
| Collagen I | ThermoFisher | Cat# A1048301 |
| Trypsin/EDTA Solution (trypsin) | ThermoFisher | Cat# R001100 |
| *Av*RII restriction enzyme | New England Biolabs | Cat# R0174S |
| *Xba*I restriction enzyme | New England Biolabs | Cat# R0145S |
| *Xho*I restriction enzyme | New England Biolabs | Cat# R0146S |
| Dimethylsulfoxid (DMSO) | Carl Roth | Cat# A994.1 |
| PF-543 | Echelon Biosciences | Cat# B-0026 |
| FTY-720-phosphate | Echelon Biosciences | Cat#B-0721-2 |
| W146 | Merck | Cat#857390P |
| JTE-013 | Merck | Cat#J4080 |
| FTY-720 | Echelon Biosciences | Cat#B-0720 |
| CYM5520 | Cayman Chemicals | Cat#17638 |
| SLM 6031434 hydrochloride | Tocris | Cat#6281 |
| K145 hydrochloride | Merck | Cat#509106 |
| CYM5442 | Cayman Chemicals | Cat#16925 |
| CYM5541 | Cayman Chemicals | Cat#15190 |
| EndoGRO Basal Medium | Merck | Cat# SCME-BM |
| Albumin (BSA) Fraction V (pH 7.0) | PanReac AppliChem | Cat# A1391 |
| Dulbecco´s Phosphate Bufferd Saline (PBS) | ThermoFisher | Cat# 21600-069 |
| Propidium iodide | Merck | Cat# 81845 |
| RNAse A | Merck | Cat# R6513 |
| Triton X-100 | Carl Roth | Cat# 3051.4 |
| Spectinomycin | Merck | Cat# S0692 |
| Erythromycin | Merck | Cat# E5389 |
| Proteose peptone | Merck | Cat# 82450 |
| Sodium hydrogen carbonate (NaHCO_3_) | Merck | Cat# 1.06329 |
| Magnesium chloride hexahydrate (MgCl_2_ x 6 H_2_O) | Carl Roth | Cat# 2189 |
| 1-Butanol ROTIPURAN ≥99,5 %, p.a., ACS | Carl Roth | Cat# 7171.1 |
| Methanol ROTIPURAN ≥99,9%, p.a., ACS, ISO | Carl Roth | Cat#4627.5 |
| Chloroform LICHROSOLV | Supelco | Cat# 1.02444 |
| 2-Mercaptoethanol | Merck | Cat# 8.05740 |
| PowerUp SYBR Green Master Mix | ThermoFisher | Cat# A25741 |
| Power SYBR Green PCR Master Mix | ThermoFisher | Cat# 4368577 |
| Ethanolamine, > 98% | Merck | Cat# E9508 |
| Ammonium sulfate | Merck | Cat# 101217 |
| Tris(hydroxymethyl)-aminomethan (TRIS), > 99,9% | Carl Roth | Cat# 5429.3 |
| Recombinant SphK1, active | Echelon Biosciences | Cat# E-K068 |
| Recombinant SphK2, active | Echelon Biosciences | Cat# E-K069 |
| Sphingosine 1-phosphate (d18:1) | Avanti Polar Lipids | Cat# 860492P |
| Sphingosine-d_7_ (d18:1) | Avanti Polar Lipids | Cat# 860657P |
| Sphingosine 1-phosphate-d_7_ (d18:1) | Avanti Polar Lipids | Cat# 860659P |
| 16:0 Ceramide (d18:1/16:0) | Avanti Polar Lipids | Cat# 860516P |
| 17:0 Ceramide (d18:1/17:0) | Avanti Polar Lipids | Cat# 860517P |
| 18:0 Ceramide (d18:1/18:0) | Avanti Polar Lipids | Cat# 860518P |
| 20:0 Ceramide (d18:1/20:0) | Avanti Polar Lipids | Cat# 860520P |
| 22:0 Ceramide (d18:1/22:0) | Avanti Polar Lipids | Cat# 860501P |
| 24:0 Ceramide (d18:1/24:0) | Avanti Polar Lipids | Cat# 860524P |
| 24:1 Ceramide (d18:1/24:1) | Avanti Polar Lipids | Cat# 860525P |
| 16:0 Sphingomyelin (d18:1/16:0) | Avanti Polar Lipids | Cat# 860584P |
| d_31_-16:0 Sphingomyelin (d18:1/16:0-d_31_) | Avanti Polar Lipids | Cat# 868584P |
| 18:0 Sphingomyelin (d18:1/18:0) | Avanti Polar Lipids | Cat# 860586P |
| 20:0 Sphingomyelin (d18:1/20:0) | Cayman Chemicals | Cat# 24450 |
| 22:0 Sphingomyelin (d18:1/22:0) | Cayman Chemicals | Cat# 24451 |
| 24:0 Sphingomyelin (d18:1/24:0) | Avanti Polar Lipids | Cat# 860592P |
| 24:1 Sphingomyelin (d18:1/24:1) | Avanti Polar Lipids | Cat# 860593P |
| Human EGF Recombinant Protein | ThermoFisher | Cat# PHG0314 |
| Hydrochloric acid, ROTIPURAN 37 %, p.a., ACS, ISO | Carl Roth | Cat# 4625.1 |
| Sodium chloride, >99%, Ph. Eur., USP | Carl Roth | Cat# P029.2 |
| EDTA | PanReac AppliChem | Cat# A1103,1000 |
| Sodium desoxycholate | Merck | Cat# 1.06504 |
| Sodium dodecyl sulphate (SDS), pellets | Carl Roth | Cat# CN30.3 |
| Sodium fluoride ACS reagent, ≥99% | Merck | Cat# 201154 |
| Halt protease & phosphatase inhibitor cocktail | ThermoFisher | Cat# 78443 |
| Glycerol, ROTIPURAN 86% , p.a. | Carl Roth | Cat# 4043.1 |
| Bromphenol blue | Merck | Cat# 1.08122 |
| Acrylamide, ROTIPHORESE Gel 30 (37.5:1) | Carl Roth | Cat# 3029.1 |
| Tween20 | Carl Roth | Cat# 9127.1 |
| Glycine | Carl Roth | Cat# 3908.3 |
| Nonfat dried milk powder (Skim Milk) | PanReac AppliChem | Cat# A0830 |
| TransIT-siQUEST Transfection Reagent | MIRUS | Cat# MIR2110 |
| Saponin | Serva Electrophoresis | Cat# 34655.02 |
| Gentamicin | ThermoFisher | Cat# 15710064 |
| Sodium carbonate | Merck | Cat# 223530 |
| Fetal calf serum (FCS) | ThrermoFisher | Cat# 10270106 |
| Sulphuric acid, ROTIPURAN≥96 %, p.a., ISO | Carl Roth | Cat# 4623.4 |
| **Critical commercial assays** | | |
| PCR Mycoplasma Test Kit | PanReac AppliChem | Cat# A3744 |
| NucleoSpin RNA, Mini kit for RNA purification | Macherey-Nagel | Cat# 740955 |
| LunaScript RT SuperMix Kit | New England Biolabs | Cat# E3010 |
| Vivaspin 20 centrifugal concentrator MWCO 5 kDa | Merck | Cat# Z614599 |
| Pierce BCA Protein Assay Kit | ThermoFisher | Cat# 23227 |
| Sphingosine Kinase Activity Assay | Echelon Biosciences | Cat# K-3500 |
| Human IL-8 ELISA Set | BD Biosciences | Cat# 555244 RRID:AB_2869050 |
| TMB substrate kit | ThermoFisher | Cat# 34021 |
| Minute Plasma Membrane Protein Isolation Kit | Invent BioTechnologies | Cat# SM-005 |
| **Experimental models: Cell lines** | | |
| hCMEC/D3 | Merck | Cat# SCC066 RRID:CVCL_U985 |
| **Oligonucleotides** | | |
| Primers for Cloning. See ***Table 1***. | This study |  |
| Primers for RT-qPCR. See ***Table 2***. | This study |  |
| Control siRNA (FITC conjugate)-A | Santa Cruz | Cat# sc-36869 |
| SPHK1 siRNA(h) | Santa Cruz | Cat# sc-44114 |
| SPHK2 siRNA(h) | Santa Cruz | Cat# sc-39225 |
| EDG-1 (S1PR1) siRNA (h) | Santa Cruz | Cat# sc-37086 |
| EDG-5 (S1PR2) siRNA (h) | Santa Cruz | Cat# sc-39928 |
| EDG-3 (S1PR3) siRNA (h) | Santa Cruz | Cat# sc-35261 |
| **Software and algorithms** | | |
| FlowJo version 10 | FlowJo, BD Biosciences | RRID:SCR_008520 |
| StepOne Software version 2.3 | ThermoFisher | RRID:SCR_014281 |
| Excel version 2032 | Microsoft | RRID:SCR_016137 |
| ImageJ | NIH | RRID:SCR_003070 |
| GraphPad Prism version 6.01 | GraphPad | RRID:SCR_002798 |
| Adobe Illustrator version 27.5 | Adobe | RRID:SCR_010279 |
| Biorender | Biorender | RRID:SCR_018361 |
| **Recombinant DNA** | | |
| pTL1 vector | Unkmeir *et al.* [[4](#_ENREF_4)] |  |
| **Other** | | |
| MACSQuant Analyzer 10 | Miltenyi Bioscience | RRID:SCR_020268 |
| Columbia agar + 5% sheep blood | bioMérieux | Cat# 43049 |
| 1290 Infinity II UHPLC coupled to 6495C triple-quadrupole mass spectrometer (LC-MS/MS) | Agilent Technologies | https://www.agilent.com |
| NanoDrop One | ThermoFisher | RRID:SCR_023005 |
| StepOnePlus | ThermoFisher | RRID:SCR_015805 |
| SpectraMax iD3 ELISA reader | Molecular Devices | https://www.moleculardevices.com/ |
| Nitrocellulose Blotting membrane 0.45 µM | Merck | Cat# GE10600002 |
| Clarity Western ECL Substrate | Bio-Rad | Cat# 170-5061 |
| ChemiDoc MP Imaging System | Bio-Rad | RRID:SCR_019037 |
| Eclipse Ti-E inverted microscope | Nikon | https://www.microscope.healthcare.nikon.com/ |
| 96-well Nunc-Immuno polystyrene Maxisorp ELISA flat bottom plates | ThermoFisher | Cat# 442404 |

# Reference

1. Tettelin H, Saunders NJ, Heidelberg J, Jeffries AC, Nelson KE, Eisen JA, et al. Complete genome sequence of Neisseria meningitidis serogroup B strain MC58. Science. 2000;287(5459):1809-15. doi: 10.1126/science.287.5459.1809. PubMed PMID: 10710307.

2. Nassif X, Beretti JL, Lowy J, Stenberg P, O'Gaora P, Pfeifer J, et al. Roles of pilin and PilC in adhesion of Neisseria meningitidis to human epithelial and endothelial cells. Proceedings of the National Academy of Sciences. 1994;91(9):3769-73. doi: 10.1073/pnas.91.9.3769.

3. Peters S, Schlegel J, Becam J, Avota E, Sauer M, Schubert-Unkmeir A. Neisseria meningitidis Type IV Pili Trigger Ca(2+)-Dependent Lysosomal Trafficking of the Acid Sphingomyelinase To Enhance Surface Ceramide Levels. Infect Immun. 2019;87(8). Epub 2019/06/05. doi: 10.1128/IAI.00410-19. PubMed PMID: 31160362; PubMed Central PMCID: PMCPMC6652772.

4. Unkmeir A, Kämmerer U, Stade A, Hübner C, Haller S, Kolb-Mäurer A, et al. Lipooligosaccharide and Polysaccharide Capsule: Virulence Factors of Neisseria meningitidis that Determine Meningococcal Interaction with Human Dendritic Cells. Infection and Immunity. 2002;70(5):2454-62. doi: 10.1128/iai.70.5.2454-2462.2002.
